# An accurate trajectory tracking method for low-speed unmanned vehicles based on model predictive control

**DOI:** 10.1038/s41598-024-60290-5

**Published:** 2024-05-10

**Authors:** Lifen Wang, Sizhong Chen, Hongbin Ren

**Affiliations:** Beijing Institute of Technology, Beijing, 100081 People’s Republic of China

**Keywords:** Electrical and electronic engineering, Mechanical engineering

## Abstract

Trajectory tracking on a low-speed vehicle using the model predictive control (MPC) algorithm usually assumes a simple road terrain. This assumption does not correspond to the actual road situation, leading to low tracking accuracy. Therefore, a trajectory tracking method considering road curvature based on MPC is proposed in this paper. In this method, the controller can automatically switch between MPC types. Linear model predictive control (LMPC) is selected for small road curvatures, while nonlinear model predictive control (NMPC) is employed for large road curvatures. In addition, the NMPC algorithm in this work considers the effect of road curvature on tracking accuracy, making it suitable for tracking time-varying curvature roads. To verify the feasibility of the algorithm, simulation comparisons with the basic MPC model were carried out at different testing roads and vehicle longitudinal speeds. The results indicate that the method significantly improves trajectory tracking accuracy, all while ensuring real-time calculations. The intelligent switching capability of control models based on road curvature allows its application to track trajectories on arbitrarily complex roads.

## Introduction

With the continuous evolution of people's demands for connected cars that can enhance driving safety and avoid traffic congestion, these vehicles have emerged as a pivotal focus in automotive advancement. As part of the research on intelligent vehicles, trajectory tracking can assist intelligent vehicles in driving stably and accurately along the planned trajectory route^1,2^. A broad spectrum of research has delved into trajectory tracking, spanning optimal control methods^3^, sliding mode control methods^4–6^, adaptive control methods^7^, robust control^8^, and fuzzy control methods^9^.

Analyzing the methods outlined in the previous paragraph allows us to discern their respective strengths and weaknesses. Optimal control, for instance addresses the original problem by breaking it down into multiple subproblems to attain a global optimal solution. However, its efficiency diminishes notably when confronted with high-dimensional or expansive state spaces. Sliding mode control boasts rapid responsiveness but entails a complex controller design. The calculations of adaptive control and robust control are simple. However, their implementation processes are intricate, and they exhibit poor real-time capabilities. Fuzzy control exhibits robustness when addressing nonlinear issues. Yet, the design of fuzzy control is not systematic enough to define the control target.

Compared to these algorithms, model predictive control (MPC) can achieve rolling optimization by predicting future states based on past states^10,11^. Additionally, it can handle multi-constraint problems by incorporating constraints into the objective function. Furthermore, MPC includes a feedback correction function^12^, which endows it with its robustness and anti-interference capabilities^13,14^, making MPC widely used in trajectory tracking. In the current research on low-speed vehicle trajectory tracking using the MPC algorithm^15,16^, a simple road terrain is usually assumed, neglecting the road curvature factor. However, due to complex geometric conditions like bending and fluctuation, limited sight lines, and various uncertain factors, the traffic accident rate in curved roads is much higher than that on ordinary roads^17^. Consequently, it is imperative to investigate trajectory tracking accuracy under complex road conditions using the MPC algorithm.

Classical MPC methods include the linear model predictive control (LMPC) and nonlinear model predictive control (NMPC) algorithms. LMPC has the advantage of simplicity in calculation and good real-time performance. However, its tracking accuracy is poor, limiting its use to predicting and controlling vehicle movement on small curvature roads or straight lines. In contrast, when unmanned vehicles operate on roads with significant curvature, NMPC^18^ is considered. Abbas has studied the feasibility of using classical NMPC for unmanned vehicle steering, yielding positive results. Nevertheless, the mathematical modeling of NMPC is very complex, and its substantial computing resource requirements can impact real-time performance^19^. Rafaila^20^ has developed a method combining LMPC and NMPC to control vehicle motion, but it lacks the capability of intelligent model selection, making it challenging to apply in the actual driving process.

To address the aforementioned issues, this research examines how road curvature and vehicle speed affect the accuracy of trajectory tracking when LMPC and NMPC are used at low vehicle speeds. The classical LMPC and NMPC methods are then selected to create a new vehicle trajectory tracking method based on road curvature. The new method significantly improves trajectory tracking accuracy, all while ensuring real-time calculations. In addition, the intelligent switching capability of control models based on road curvature allows its application to track trajectories on arbitrarily complex roads in the actual driving process.

The remainder of the article is structured as follows: Section "Trajectory tracking method based on MPC" introduces the trajectory tracking method based on MPC, Section "Simulations and analysis" presents the simulation comparisons between the proposed method and the basic MPC model at different testing roads and vehicle longitudinal speeds, and Section "Real-time performance" gives the results of this paper.

## Trajectory tracking method based on MPC

### Preliminaries of MPC

Many studies have been conducted on trajectory tracking based on MPC^15,16^. Moreover, some scholars have compared the tracking performance of the MPC algorithm with other algorithms. For instance, Kai Yang^21^ compared MPC with the Robust H-infinity State Feedback Control in Trajectory Tracking, demonstrating that the MPC shows better tracking accuracy and response time. Duoyang Qiu^22^ designed a controller based on MPC to track parking trajectories, and the results indicate that the designed controller achieves better tracking accuracy compared to traditional PID controllers. In this paper, a trajectory tracking method that considers road curvature based on MPC is proposed.

**Figure 1:**
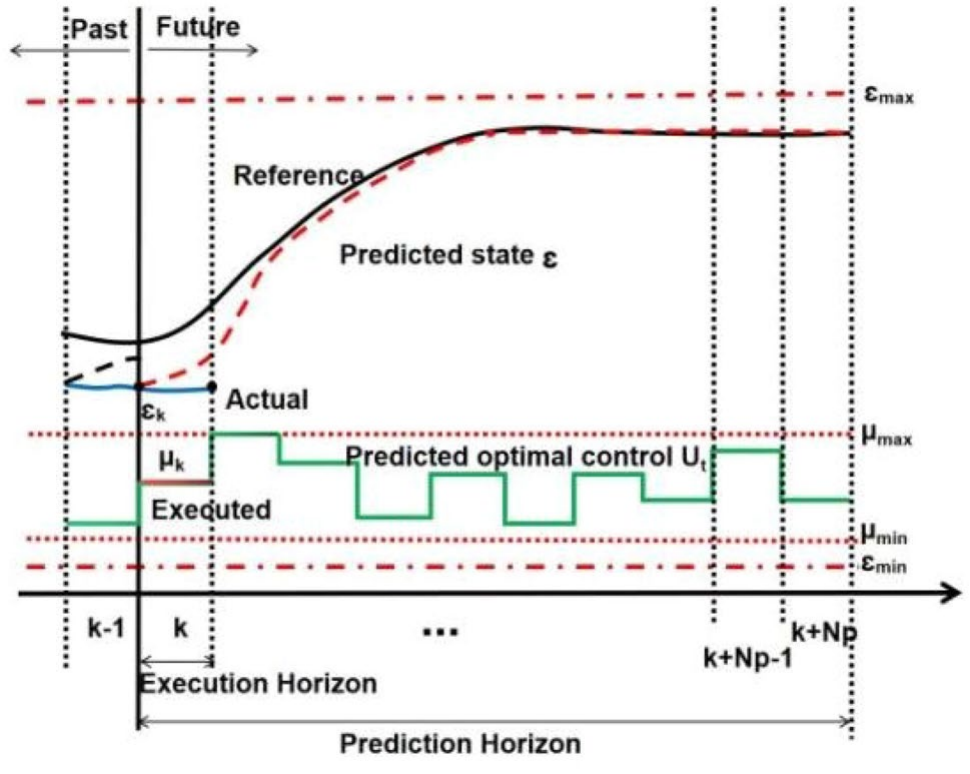
Basic principle of MPC.

As illustrated in Fig. 1, MPC is implemented by iterative online optimization across a moving finite prediction horizon. To derive the future system dynamics at the k-th time step, input parameters including state and control variables are defined as the initial parameters. A finite-time and constrained optimal control problem is calculated online in an open-loop condition over the prediction horizon Np. While a control sequence is optimized over the prediction horizon Np, only the first component u_k_ is executed to obtain the system output Ɛ_k+1_, ensuring that the output closely approaches the reference value Ɛ_r_ at k + 1.

The optimal process is repeated in the subsequent steps based on the new input until the terminal requirements are satisfied.

### Vehicle kinematic model

**Figure 2:**
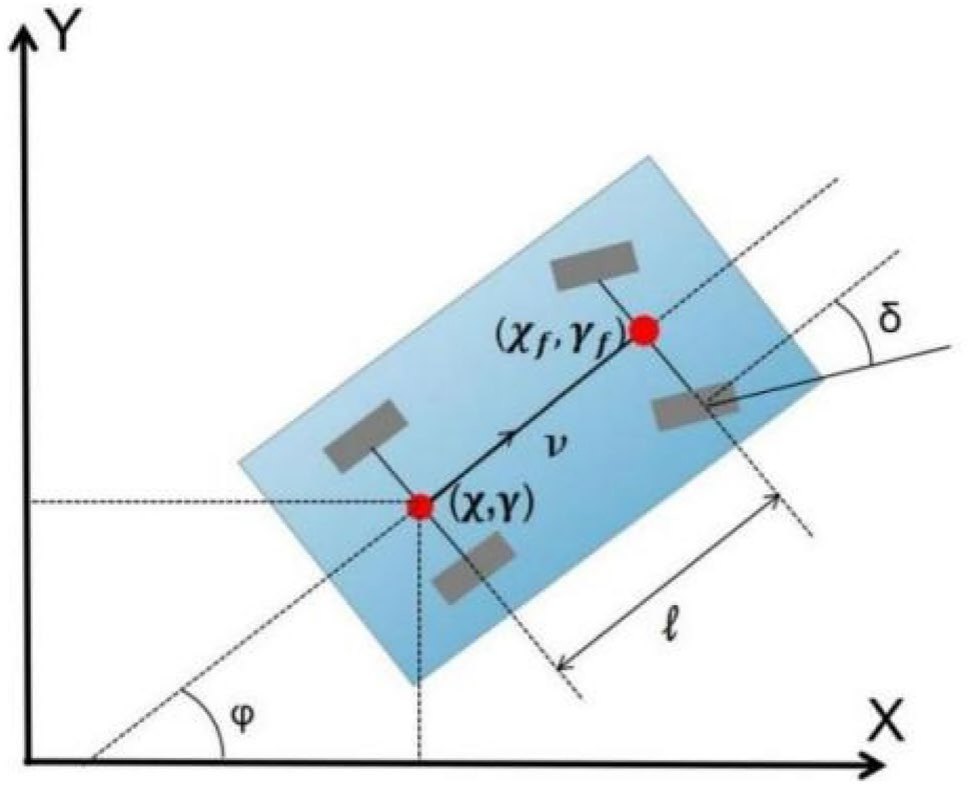
Vehicle kinematics diagram.

Figure 2 depicts a typical two-degree-of-freedom (2-DOF) vehicle steering kinematic model, with ($\chi_{f}$*,*$\gamma_{f}$) denoting the front axle center position while (*x, y*) denotes its rear position,* δ* represents the front wheel angle, *φ* represents the yaw angle for the vehicle, $\nu$ denotes the rear axle center speed, and *ℓ* represents the vehicle wheelbase.

At the driving center of the rear axle, the speed $\nu$ can be expressed as:

1 $$\nu = \dot{\chi }\cos \varphi + \dot{\gamma }\sin \varphi$$

The kinematic constraints of the front and rear axles are given by:

2 $$\dot{\chi }_{f} \sin \left( {\varphi + \delta } \right) - \dot{\gamma }_{f} \cos \left( {\varphi + \delta } \right) = 0$$

3 $$\dot{\chi }\sin \varphi + \dot{\gamma }\cos \varphi = 0$$

According to Eqs. (1–3), we can obtain:

4 $$\dot{\chi } = \nu \cos \varphi$$

5 $$\dot{\gamma } = \nu \sin \varphi$$

The following is derived from the geometric relationship between the front and rear wheels:

6 $$\chi_{f} = \chi + l\cos \varphi$$

7 $$\gamma_{f} = \gamma + l\sin \varphi$$

Substituting Eqs. (4–7) into Eqs. (2, 3), we can solve for the yaw velocity as:

8 $$\omega = \frac{\nu }{l}\tan \varphi_{f}$$

Another way to express the yaw velocity:

9 $$\omega = \dot{\varphi }$$

Thus, the kinematics model for Fig. 2 can be expressed as:

10 $$\left\{ {\begin{array}{*{20}c} {\dot{\chi } = \nu_{\chi } = \nu \cos \varphi } \\ {\dot{\gamma } = \nu_{\gamma } = \nu \sin \varphi } \\ {\dot{\varphi } = \frac{\nu \tan \delta }{{\text{l}}}} \\ \end{array} } \right.$$

### Model predictive control

#### Linear model predictive control

Because of its simplicity in calculation and good real-time performance, LMPC has been used in trajectory tracking over the past few years^23,24^. This section outlines the process of linear discretization for the vehicle kinematics model to construct the LMPC problem^25^.

In the trajectory tracking control process, the control and state quantities can be expressed as Eq. (11) based on Eq. (10):

11 $$\dot{\varepsilon } = f\left( {\varepsilon ,\mu } \right)$$

where $\varepsilon$=${\left[x,y,\varphi \right]}^{{\text{T}}}$ and $u$=${\left[\upsilon ,\delta \right]}^{{\text{T}}}$. Using Taylor expansion at the reference point and keeping only the first order terms, the model can be expressed as:

12 $$\dot{\varepsilon } = f\left( {\varepsilon_{r} ,\mu {}_{r}} \right) + \frac{\partial f}{{\partial \varepsilon }}\left| {\begin{array}{*{20}c} {\varepsilon = \varepsilon_{r} } \\ {\mu = \mu_{r} } \\ \end{array} } \right.\left( {\varepsilon - \varepsilon_{{\text{r}}} } \right) + \frac{\partial f}{{\partial \mu }}\left| {\begin{array}{*{20}c} {\varepsilon = \varepsilon_{r} } \\ {\mu = \mu_{r} } \\ \end{array} } \right.\left( {\mu - \mu_{{\text{r}}} } \right)$$

where $$\frac{\partial f}{{\partial \varepsilon }}\left| {\begin{array}{*{20}c} {\varepsilon = \varepsilon_{r} } \\ {\mu = \mu_{r} } \\ \end{array} } \right. = \left[ {\begin{array}{*{20}l} {\frac{{\partial f_{1} }}{{\partial \chi_{r} }}} \hfill & {\quad \frac{{\partial f_{1} }}{{\partial \gamma_{r} }}} \hfill & {\quad \frac{{\partial f_{1} }}{{\partial \varphi_{r} }}} \hfill \\ {\frac{{\partial f_{2} }}{{\partial \chi_{r} }}} \hfill & {\quad \frac{{\partial f_{2} }}{{\partial \gamma_{r} }}} \hfill & {\quad \frac{{\partial f_{2} }}{{\partial \varphi_{r} }}} \hfill \\ {\frac{{\partial f_{3} }}{{\partial \chi_{r} }}} \hfill & {\quad \frac{{\partial f_{3} }}{{\partial \gamma_{r} }}} \hfill & {\quad \frac{{\partial f_{3} }}{{\partial \varphi_{r} }}} \hfill \\ \end{array} } \right] = \left[ {\begin{array}{*{20}l} 0 \hfill & {\quad 0} \hfill & {\quad - \nu_{r} \sin \varphi_{r} } \hfill \\ 0 \hfill & {\quad 0} \hfill & {\quad \nu_{r} \cos \varphi_{r} } \hfill \\ 0 \hfill & {\quad 0} \hfill & {\quad 0} \hfill \\ \end{array} } \right]$$,$$\frac{{\partial {\text{f}}}}{\partial \mu }\left| {\begin{array}{*{20}c} {\varepsilon = \varepsilon_{r} } \\ {\mu = \mu_{r} } \\ \end{array} } \right. = \left[ {\begin{array}{*{20}l} {\frac{{\partial f_{1} }}{{\partial \nu_{r} }}} \hfill & {\quad \frac{{\partial f_{1} }}{{\partial \delta_{r} }}} \hfill \\ {\frac{{\partial f_{2} }}{{\partial \nu_{r} }}} \hfill & {\quad \frac{{\partial f_{2} }}{{\partial \delta_{r} }}} \hfill \\ {\frac{{\partial f_{3} }}{{\partial \nu_{r} }}} \hfill & {\quad \frac{{\partial f_{3} }}{{\partial \delta_{r} }}} \hfill \\ \end{array} } \right] = \left[ {\begin{array}{*{20}l} \begin{gathered} \cos \varphi_{r} \hfill \\ \sin \varphi_{r} \hfill \\ \tfrac{{\tan \varphi_{r} }}{{\text{l}}} \hfill \\ \end{gathered} \hfill & \begin{gathered} \quad 0 \\ \quad 0 \\ \quad \tfrac{{\nu_{r} }}{{{\text{l}}\cos^{2} \delta_{r} }} \\ \end{gathered} \hfill \\ \end{array} } \right]$$, and $\varepsilon_{{\text{r}}}$, $\mu_{{\text{r}}}$ are the referenced state quantity and control quantity, respectively.

The change in the state quantity error can be expressed as:

13 $$\dot{\tilde{\varepsilon }} = \left[ {\begin{array}{*{20}c} {\dot{\chi } - \dot{\chi }_{r} } \\ {\dot{\gamma } - \dot{\gamma }_{r} } \\ {\dot{\varphi } - \dot{\varphi }_{r} } \\ \end{array} } \right] = \frac{\partial f}{{\partial \varepsilon }}\left| {_{{(\varepsilon - \varepsilon_{r} )}} } \right. + \frac{\partial f}{{\partial \mu }}\left| {_{{(\mu - \mu_{r} )}} } \right. = \left[ {\begin{array}{*{20}l} 0 \hfill & {\quad 0} \hfill & {\quad - \nu_{r} \sin \varphi_{r} } \hfill \\ 0 \hfill & {\quad 0} \hfill & {\quad - \nu_{r} \cos \varphi_{r} } \hfill \\ 0 \hfill & {\quad 0} \hfill & {\quad 0} \hfill \\ \end{array} } \right]\left[ {\begin{array}{*{20}c} {\chi - \chi_{r} } \\ {\gamma - \gamma_{r} } \\ {\varphi - \varphi_{r} } \\ \end{array} } \right] + \left[ {\begin{array}{*{20}l} \begin{gathered} \cos \varphi_{r} \hfill \\ \sin \varphi_{r} \hfill \\ \tfrac{{\tan \varphi_{r} }}{l} \hfill \\ \end{gathered} \hfill & \begin{gathered} \quad 0 \\ \quad 0 \\ \quad \tfrac{{\nu_{r} }}{{l\cos^{2} \delta_{r} }} \\ \end{gathered} \hfill \\ \end{array} } \right]\left[ {\begin{array}{*{20}c} {\nu - \nu_{r} } \\ {\delta - \delta_{r} } \\ \end{array} } \right] = A\tilde{\varepsilon } + B\tilde{\mu }$$

where $${\text{A}} = \left[ {\begin{array}{*{20}l} 0 \hfill & {\quad 0} \hfill & {\quad - \nu_{{\text{r}}} \sin \varphi_{r} } \hfill \\ 0 \hfill & {\quad 0} \hfill & {\quad \nu_{{\text{r}}} \cos \varphi_{r} } \hfill \\ 0 \hfill & {\quad 0} \hfill & {\quad 0} \hfill \\ \end{array} } \right]$$, and $$B = \left[ {\begin{array}{*{20}l} \begin{gathered} \cos \varphi_{r} \hfill \\ \sin \varphi_{r} \hfill \\ \tfrac{{\tan \varphi_{r} }}{l} \hfill \\ \end{gathered} \hfill & \begin{gathered} \quad 0 \\ \quad 0 \\ \quad \tfrac{{\nu_{r} }}{{l\cos^{2} \delta_{r} }} \\ \end{gathered} \hfill \\ \end{array} } \right].$$

The Euler method is consistently used here for model discretization. Given the computational intensity of backward Euler^26^, we opt for forward Euler to formulate the MPC problem. Upon applying forward Euler discretization to Eq. (13):

14 $$\dot{\tilde{\varepsilon }} = \frac{{\tilde{\varepsilon }(k + 1) - \tilde{\varepsilon }(k)}}{T} = A\tilde{\varepsilon } + B\tilde{\mu }$$

The transformation of Eq. (14) is:

15 $$\tilde{\varepsilon }(k + 1) = (IA + E)\tilde{\varepsilon }(k) + TB\tilde{\mu }(k) = \tilde{A}\tilde{\varepsilon }(k) + \tilde{B}\tilde{\mu }(k)$$

where *I* is the identity matrix, $${\tilde{\text{A}}} = \left[ {\begin{array}{*{20}l} 1 \hfill & {\quad 0} \hfill & {\quad - \nu_{{\text{r}}} \sin \varphi_{r} } \hfill \\ 0 \hfill & {\quad 1} \hfill & {\quad \nu_{{\text{r}}} \cos \varphi_{r} } \hfill \\ 0 \hfill & {\quad 0} \hfill & {\quad 1} \hfill \\ \end{array} } \right]$$, and $$\tilde{B} = \left[ {\begin{array}{*{20}l} \begin{gathered} T\cos \varphi_{r} \hfill \\ T\sin \varphi_{r} \hfill \\ T\tfrac{{\tan \varphi_{r} }}{l} \hfill \\ \end{gathered} \hfill & \begin{gathered} \quad 0 \\ \quad 0 \\ \quad T\tfrac{{\nu_{r} }}{{l\cos^{2} \delta_{r} }} \\ \end{gathered} \hfill \\ \end{array} } \right].$$

By assigning $\widetilde{A}$ to a and $\widetilde{B}$ to b, Eq. (15) can be expressed as:

16 $$\tilde{\varepsilon }\left( {k + 1} \right) = a\tilde{\varepsilon }\left( k \right) + b\tilde{\mu }\left( k \right)$$

where $$\tilde{\varepsilon } = \left[ {\begin{array}{*{20}c} {\chi - \chi_{{\text{r}}} } \\ {\gamma - \gamma_{r} } \\ {\varphi - \varphi_{r} } \\ \end{array} } \right]$$* and *$$\tilde{\mu } = \left[ {\begin{array}{*{20}c} {\nu - \nu_{{\text{r}}} } \\ {\delta - \delta_{r} } \\ \end{array} } \right]$$*.*

The output equation can be defined as:

17 $$\gamma \left( k \right) = \left[ {\begin{array}{*{20}c} 1 & {\quad 0} & {\quad 0} \\ 0 & {\quad 1} & {\quad 0} \\ 0 & {\quad 0} & {\quad 1} \\ \end{array} } \right]\tilde{\varepsilon }\left( k \right) = C\tilde{\varepsilon }\left( k \right)$$

where $$C = \left[ {\begin{array}{*{20}c} 1 & {\quad 0} & {\quad 0} \\ 0 & {\quad 1} & {\quad 0} \\ 0 & {\quad 0} & {\quad 1} \\ \end{array} } \right]$$.

The cost function should be able to ensure that the unmanned vehicle can track the desired trajectory quickly and smoothly. Therefore, it is necessary to add the state quantity deviation and the control quantity into the cost function. When designing the trajectory tracking controller, the following cost function is used:

18 $$J = \sum\limits_{j = 1}^{N} {\mathop {\tilde{\varepsilon }}\nolimits^{T} \left( {k + j} \right)} Q\tilde{\varepsilon }\left( {k + j} \right) + \mathop {\tilde{\mu }}\nolimits^{T} \left( {k + j - 1} \right)R\tilde{\mu }\left( {k + j - 1} \right)$$

where Q and R are weigh matrices.

This objective function cannot limit the control increment in each sampling period, potentially causing sudden changes in control quantity and leading to its discontinuity. To overcome this limitation, a new state quantity is built:

19 $$\xi \left( {\text{k}} \right) = \left[ {\begin{array}{*{20}c} {\tilde{\varepsilon }\left( k \right)} \\ {\tilde{\mu }\left( {k - 1} \right)} \\ \end{array} } \right]$$

The new state space is expressed as:

20 $$\begin{gathered} \xi \left( {{\text{k}} + 1} \right) = \left[ {\begin{array}{*{20}l} {\tilde{\varepsilon }\left( {k + 1} \right)} \hfill \\ {\tilde{\mu }\left( k \right)} \hfill \\ \end{array} } \right] = \left[ {\begin{array}{*{20}l} {a\tilde{\varepsilon }\left( k \right) + b\tilde{\mu }\left( k \right)} \hfill \\ {\tilde{\mu }\left( k \right)} \hfill \\ \end{array} } \right] = \left[ {\begin{array}{*{20}l} {a\tilde{\varepsilon }\left( k \right) + b\tilde{\mu }\left( {k - 1} \right) + b\tilde{\mu }\left( k \right) - b\tilde{\mu }\left( {k - 1} \right)} \hfill \\ {\tilde{\mu }\left( {k - 1} \right) + \tilde{\mu }\left( k \right) - \tilde{\mu }\left( {k - 1} \right)} \hfill \\ \end{array} } \right] \\ = \left[ {\begin{array}{*{20}l} {\left[ {\begin{array}{*{20}c} {\text{a}} & b \\ \end{array} } \right]} \hfill & {\left[ {\begin{array}{*{20}c} {\tilde{\varepsilon }\left( k \right)} \\ {\tilde{\mu }\left( {k - 1} \right)} \\ \end{array} } \right]} \hfill \\ {\left[ {\begin{array}{*{20}c} o & {I_{Nu} } \\ \end{array} } \right]} \hfill & {\left[ {\begin{array}{*{20}c} {\tilde{\varepsilon }\left( k \right)} \\ {\tilde{\mu }\left( {k - 1} \right)} \\ \end{array} } \right]} \hfill \\ \end{array} } \right] + \left[ {\begin{array}{*{20}c} b \\ {I_{Nu} } \\ \end{array} } \right]\left( {\tilde{\mu }\left( k \right) - \tilde{\mu }\left( {k - 1} \right)} \right) = \left[ {\begin{array}{*{20}l} {\text{a}} \hfill & {\quad b} \hfill \\ o \hfill & {\quad I_{Nu} } \hfill \\ \end{array} } \right]\xi \left( k \right) + \left[ {\begin{array}{*{20}c} b \\ {I_{Nu} } \\ \end{array} } \right]\Delta \tilde{\mu }\left( k \right) \\ = \hat{A}\xi \left( k \right) + \hat{B}\Delta \tilde{\mu }\left( k \right) \\ \end{gathered}$$

where $$\hat{A} = \left[ {\begin{array}{*{20}l} a \hfill & {\quad b} \hfill \\ o \hfill & {\quad I_{Nu} } \hfill \\ \end{array} } \right]$$ and $${\hat{\text{B}}} = \left[ {\begin{array}{*{20}c} b \\ {I_{Nu} } \\ \end{array} } \right]$$.

The new output equation can be defined as:

21 $$\eta \left( {\text{k}} \right) = \left[ {\begin{array}{*{20}c} {{\text{I}}_{{{\text{Nx}}}} } & {\quad o} \\ \end{array} } \right]\left[ {\begin{array}{*{20}l} {\tilde{\varepsilon }\left( k \right)} \hfill \\ {\tilde{\mu }\left( {k - 1} \right)} \hfill \\ \end{array} } \right] = C\xi \left( k \right)$$

where ${I}_{Nx}$ is an identity matrix with N${\text{x}}$ dimension. From Eq. (20) and Eq. (21), the output quantities can be calculated as follows:

22 $${\text{Y}} = \psi \xi \left( k \right) + \Theta \Delta \mu$$

where $${\text{Y}} = \left[ {\begin{array}{*{20}c} {\eta \left( {k + 1} \right)} \\ {\eta \left( {k + 2} \right)} \\ {\eta \left( {k + 3} \right)} \\ \vdots \\ {\eta \left( {k + Nc} \right)} \\ {\eta \left( {k + Np} \right)} \\ \end{array} } \right]$$, $$\psi = \left[ {\begin{array}{*{20}c} {{\text{CA}}} \\ {{\text{CA}}^{{2}} } \\ {{\text{CA}}^{{3}} } \\ \vdots \\ {{\text{CA}}^{{{\text{Nc}}}} } \\ {{\text{CA}}^{{{\text{Np}}}} } \\ \end{array} } \right]$$, $$\xi \left( {\text{k}} \right) = \left[ {\begin{array}{*{20}c} {\tilde{\varepsilon }\left( k \right)} \\ {\tilde{\mu }\left( {k - 1} \right)} \\ \end{array} } \right]$$, $$\Theta = \left[ {\begin{array}{*{20}l} \begin{gathered} {\text{CB}} \\ {\text{CAB}} \\ \cdots \\ {\text{CA}}^{{{\text{Nc - }}1}} {\text{B}} \\ \cdots \\ {\text{CA}}^{{{\text{Np - }}1}} {\text{B}} \\ \end{gathered} \hfill & \begin{gathered} \quad 0 \\ \quad {\text{CB}} \\ \quad \cdots \\ \quad {\text{CA}}^{{{\text{Nc - }}2}} {\text{B}} \\ \quad \cdots \\ \quad {\text{CA}}^{{{\text{Np - }}2}} {\text{B}} \\ \end{gathered} \hfill & \begin{gathered} \quad 0 \\ \quad 0 \\ \quad \cdots \\ \quad {\text{CA}}^{{{\text{Nc - }}3}} {\text{B}} \\ \quad \cdots \\ \quad {\text{CA}}^{{{\text{Np - }}3}} {\text{B}} \\ \end{gathered} \hfill & \begin{gathered} \quad \cdots \\ \quad \cdots \\ \quad \ddots \\ \quad \cdots \\ \quad \ddots \\ \quad \cdots \\ \end{gathered} \hfill & \begin{gathered} \quad 0 \\ \quad 0 \\ \quad 0 \\ \quad {\text{CA}}^{0} {\text{B}} \\ \quad \cdots \\ \quad {\text{CA}}^{{{\text{Np - }}Nc}} {\text{B}} \\ \end{gathered} \hfill \\ \end{array} } \right]$$ and $$\Delta \mu = \left[ {\begin{array}{*{20}l} {\Delta \tilde{\mu }\left( {\text{k}} \right)} \hfill \\ {\Delta \tilde{\mu }\left( {{\text{k + }}1} \right)} \hfill \\ {\Delta \tilde{\mu }\left( {{\text{k + }}2} \right)} \hfill \\ \vdots \hfill \\ {\Delta \tilde{\mu }\left( {{\text{k + }}Nc - 1} \right)} \hfill \\ \end{array} } \right]$$.

The optimization objective function can be transformed to:

23 $$J = \sum\limits_{i = 1}^{Np} {\left\| {\eta (k + i) - \eta_{r} (k + i)} \right\|_{Q}^{2} + } \sum\limits_{i = 1}^{{N{\text{c - }}1}} {\left\| {\Delta \mu (k + i)} \right\|_{{\text{R}}}^{2} } + \rho \lambda^{2}$$

Here $\lambda$ is the relaxation factor.

Define the system output reference values as:

24 $$Yr = \left[ {\begin{array}{*{20}c} {\eta_{r} (k + 1)} & {\eta_{r} (k + 2)} & \ldots & {\begin{array}{*{20}c} {\eta_{r} (k + Nc)} & \ldots & {\eta_{r} (k + Np)} \\ \end{array} } \\ \end{array} } \right]^{T} = \left[ {\begin{array}{*{20}c} 0 & 0 & \ldots & {\begin{array}{*{20}c} 0 & \ldots & 0 \\ \end{array} } \\ \end{array} } \right]^{T}$$

Let ${\text{E}} = \psi \xi \left( k \right)$, $Q = I_{Np} \otimes Q$, and $R = I_{Np} \otimes R$, then:

25 $$J = (E + \Theta \Delta \mu )^{T} Q(E + \Theta \Delta \mu ) + \Delta \mu^{T} R\Delta \mu + \rho \lambda^{2}$$

Simplify Eq. (25) as:

26 $$J = E^{T} QE + \Delta \mu^{T} (\Theta^{T} Q\Theta + R)\Delta \mu + 2E^{T} Q\Theta \Delta \mu + \rho \lambda^{2}$$

Since $E^{T} QE$ is independent of $\Delta \mu$, it can be neglected.

Then the objective function can be transformed into a quadratic form in quadprog:

27 $$J = \left[ {\begin{array}{*{20}c} {\Delta \mu^{T} } & \lambda \\ \end{array} } \right]\left[ {\begin{array}{*{20}c} {\Theta^{T} Q\Theta + R} & 0 \\ 0 & \rho \\ \end{array} } \right]\left[ {\begin{array}{*{20}c} {\Delta \mu^{T} } & \lambda \\ \end{array} } \right]^{{\text{T}}} + \left[ {\begin{array}{*{20}c} {2E^{T} Q\Theta } & 0 \\ \end{array} } \right]\left[ {\begin{array}{*{20}c} {\Delta \mu^{T} } & \lambda \\ \end{array} } \right]^{{\text{T}}}$$

According to Ye^27^, under low-speed working conditions, the disparity in tracking accuracy between the kinematic and dynamic models is small. In this paper, we use the kinematic model, which doesn’t account for tire slip angle, to construct the MPC controller. Thus, the sideslip angle is not constrained here. The following constraints for control quantity and control increment are introduced to the system to meet the actual operation requirements:

28 $${\tilde{\text{U}}}_{\min } \le {\tilde{\text{U}}} \le {\tilde{\text{U}}}_{{{\text{max}}}}$$

29 $$\Delta \tilde{\mu }_{\min } \le \Delta \tilde{\mu } \le \Delta \tilde{\mu }_{\max }$$

where $${\tilde{\text{U}}} = \left[ {\begin{array}{*{20}l} {\tilde{\mu }\left( k \right)} \hfill \\ {\tilde{\mu }\left( {k + 1} \right)} \hfill \\ \cdots \hfill \\ {\tilde{\mu }\left( {k + Nc - 1} \right)} \hfill \\ \end{array} } \right]$$, $$\Delta \tilde{\mu } = \left[ {\begin{array}{*{20}l} {\Delta \tilde{\mu }\left( k \right)} \hfill \\ {\Delta \tilde{\mu }\left( {k + 1} \right)} \hfill \\ \cdots \hfill \\ {\Delta \tilde{\mu }\left( {k + Nc - 1} \right)} \hfill \\ \end{array} } \right]$$.

In this paper, $${\tilde{\text{U}}}_{\min } = \left[ {\begin{array}{*{20}c} { - 0.2} & { - 0.436} \\ \end{array} } \right]$$, $${\tilde{\text{U}}}_{\max } = \left[ {\begin{array}{*{20}c} {0.2} & {0.436} \\ \end{array} } \right]$$, $$\Delta \tilde{\mu }_{\min } = \left[ {\begin{array}{*{20}c} { - 0.05} & { - 0.0082} \\ \end{array} } \right]$$, $$\Delta \tilde{\mu }_{\max } = \left[ {\begin{array}{*{20}c} {0.05} & {0.0082} \\ \end{array} } \right]$$.

The parameters in the above formulas are described in Table 1.

**Table 1:** Employed model parameters.

Parameters	Description	Parameters	Description
$\varepsilon$	State quantity	$\Delta \tilde{\mu }_{\min }$	The allowed minimum $\Delta \tilde{\mu }$
$u$	Control quantity	${\tilde{\text{U}}}$	The control quantity error matrix in the control domain
$\varepsilon$_*r*_	Referenced state quantity	${\tilde{\text{U}}}_{\max }$	The allowed maximum ${\tilde{\text{U}}}$
$u$_*r*_	Referenced control quantity	${\tilde{\text{U}}}_{\min }$	The allowed minimal ${\tilde{\text{U}}}$
*A*	Partial derivative of f with respect to x	$\lambda$	The relaxation coefficient
*B*	Partial derivative of f with respect to ${\text{u}}$	$\xi$*(k)*	New state quantity
$\widetilde{A}$	Discrete A	*η(k)*	Output quantity in the predict domain
$\widetilde{B}$	Discrete B	*C*	Selection matrix
*T*	Sample time interval	*Y*	Output quantities matrix
*I*	Unit matrix	*Y*_*r*_	Referenced output matrix
$\widetilde{\varepsilon }$	State quantity error	*Q, R*	Weight matrices
$\widetilde{u}$	Control quantity error	$Np$	Predictions horizon
$\Delta \tilde{\mu }$	The increment of $\widetilde{u}$	$Nc$	Control horizon
$\Delta \tilde{\mu }_{\max }$	The allowed maximum $\Delta \tilde{\mu }$		

#### Nonlinear model predictive control

Due to its high tracking accuracy, NMPC has been applied in various fields for trajectory tracking^28^. However, this NMPC is based on the vehicle kinematics model, where the impact of curvature is not considered. This can lead to a reduction in tracking accuracy when the curvature is large. To solve this problem, this paper designs a NMPC model based on tracking errors, which can impose constraints on heading angle deviation and distance deviation. At the same time, it can consider the influence of road curvature on the cost function, which is conducive to improving the tracking effect on roads with large curvature. The construction process of NMPC based on tracking errors is outlined in the sequel.

**Figure 3:**
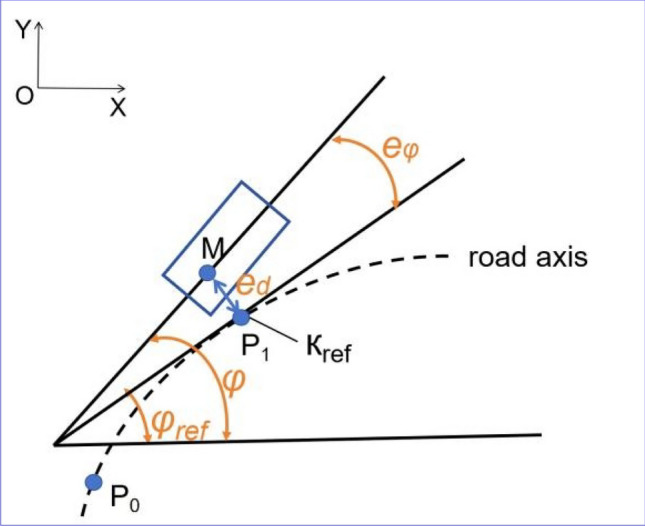
Vehicle tracking error model.

Figure 3 shows the diagram of tracking error model, and P_1_ is the projection of the vehicle’s rear axle center M on the road center line. It is assumed that the instantaneous turning radius of the vehicle is the same as the curvature radius of the road. Therefore, the reference curvature at P_1_ can be denoted as:

30 $$\kappa_{{{\text{ref}}}} = 1/R$$

*where R* is the steering radius of vehicle’s rear wheel.

Under the condition that the angular velocity of point M is the same as that of point P_1_, the following relation can be obtained:

31 $$\omega = \dot{S}/R = \left[ {\nu_{\chi } \cos \left( {e_{\varphi } } \right) - \nu_{y} \sin \left( {e_{\varphi } } \right)} \right]/\left( {R - e_{d} } \right)$$

where S is the arc length between P1 and P0.

Substituting Eq. (30) into Eq. (31), ${\dot{\text{S}}}$ can be expressed as:

32 $$\dot{S} = \frac{1}{{1 - \kappa_{ref} e_{d} }}\left[ {\nu_{\chi } \cos \left( {e_{\varphi } } \right) - \nu_{\gamma } \sin \left( {e_{\varphi } } \right)} \right]$$

It can be seen from Fig. 3 that:

33 $$\left\{ {\begin{array}{*{20}l} {{\text{e}}_{\varphi } = \varphi - \varphi_{road} } \hfill \\ {\dot{e}_{d} = \nu_{\chi } {\text{sin}}\left( {{\text{e}}_{\varphi } } \right) + \nu_{\gamma } \cos \left( {{\text{e}}_{\varphi } } \right)} \hfill \\ \end{array} } \right.$$

Therefore, the vehicle tracking error equations can be expressed as:

34 $$\left\{ {\begin{array}{*{20}l} {\dot{e}_{\varphi } = \dot{\varphi } - \kappa_{{{\text{ref}}}} {\dot{\text{S}}}} \hfill \\ {\dot{e}_{d} = \nu_{\chi } {\text{sin}}\left( {{\text{e}}_{\varphi } } \right) + \nu_{\gamma } \cos \left( {{\text{e}}_{\varphi } } \right)} \hfill \\ \end{array} } \right.$$

Assuming that the heading angle deviation is small, then ${\text{sin}}\left( {{\text{e}}_{\varphi } } \right) \approx 0$, ${\text{cos}}\left( {{\text{e}}_{\varphi } } \right) \approx 1$.

In addition, through the assumption that the tracking error is small , $\kappa_{{{\text{ref}}}} e_{d} \approx 0$ can also be obtained.

As a result, Eq. (34) can be simplified as:

35 $$\left\{ {\begin{array}{*{20}l} {\dot{e}_{\varphi } = \dot{\varphi } - \frac{{\kappa_{{{\text{ref}}}} \nu_{\chi } }}{{1 - \kappa_{{{\text{ref}}}} {\text{e}}_{d} }} \approx \dot{\varphi } - \kappa_{{{\text{ref}}}} \nu_{\chi } } \hfill \\ {\dot{e}_{d} = \nu_{\chi } {\text{e}}_{\varphi } + \nu_{\gamma } } \hfill \\ \end{array} } \right.$$

When ignoring the vehicle’s lateral speed $\nu_{\gamma }$, Eq. (36) can be obtained.

36 $$\dot{\varphi } = \omega = \frac{{\nu_{\chi } }}{{\text{R}}} = \kappa \nu_{\chi }$$

Thus, Eq. (35) can be expressed as:

37 $$\left\{ {\begin{array}{*{20}l} {\dot{e}_{\varphi } = \left( {\kappa - \kappa_{{{\text{ref}}}} } \right)\nu_{\chi } } \hfill \\ {\dot{e}_{d} = \nu_{\chi } {\text{e}}_{\varphi } } \hfill \\ \end{array} } \right.$$

where $$\varepsilon = \left[ {{\text{e}}_{\varphi } ,e_{d} } \right]$$, $$\mu = \left[ {\nu_{\chi } ,\kappa } \right]^{{\text{T}}}$$*.*

To ensure tracking and control accuracy, we construct the cost function as follows:

38 $$J = (\varepsilon - \varepsilon_{ref} )^{T} Q(\varepsilon - \varepsilon_{ref} ) + (\mu - \mu_{ref} )^{T} P(\mu - \mu_{ref} ) + \lambda \rho^{2}$$

where $\varepsilon = \left[ {{\text{e}}_{\varphi } ,e_{d} } \right]$ is the predicted state quantity under the control quantity $\mu = \left[ {\nu_{\chi } ,\kappa } \right]^{{\text{T}}}$, $\varepsilon_{{{\text{ref}}}}$ represents the expected state quantity, $\mu_{{{\text{ref}}}}$ represents the expected control quantity which includes vehicle velocity and road curvature, *Q* is the state quantitative weight and *P* represents the control quantity weight, $\rho = \left[ {\Delta X,\Delta Y} \right]^{{\text{T}}}$ represents the relaxing factor and $\lambda$ is the relaxing coefficient.

39 $$\left\{ \begin{gathered} \Delta X_{{{\text{k}},t}} = T*\nu_{\chi } *\cos \varphi = T*\nu_{\chi } *\cos \left( {e_{\varphi } + \varphi_{ref} } \right) \hfill \\ \Delta Y_{{{\text{k}},t}} = T*\nu_{\chi } *\sin \varphi = T*\nu_{\chi } *\sin \left( {e_{\varphi } + \varphi_{ref} } \right) \hfill \\ \end{gathered} \right.$$

$\Delta {\text{X}}_{{\text{k,t}}}$ is the component of the vehicle’s longitudinal path increment in the X-axis of the inertial coordinate at the k-th time step. $\Delta {\text{Y}}_{{\text{k,t}}}$ is the component of the vehicle’s longitudinal path increment in the Y-axis of the inertial coordinate at k-th time step.

Thus, nonlinear model predictive control can be realized by solving the following nonlinear minimum optimization with constraints in Eq. (41)–(43):

min $$J(\varepsilon_{t} ,\mu_{t} )$$

40 $${\text{s}}.t.\quad \varepsilon_{k + 1,t} = f(\varepsilon_{k,t} ,\mu_{k,t} ),\;{\text{k}} = t, \ldots ,N - 1$$

41 $$\varepsilon_{k,t} \in I,\;k = t, \cdots ,t + N - 1,$$

42 $$\mu_{k,t} \in \Gamma ,\;k = t, \ldots ,t + N - 1,$$

43 $${\text{E}} \le \mu_{k,t} - \mu_{{{\text{ref}}}} \le H,\;{\text{k}} = t, \ldots ,{\text{t}} + N - 1,$$

Here, Eq. (41) denotes the state constraint, while Eqs. (42)-(43) are the constraint for control quantities.

In this paper,$${\text{I}} = \left[ {\begin{array}{*{20}l} {\left[ { - 0.24, 0.24} \right]} \hfill \\ {\left[ { - 0.7, 0.7} \right]} \hfill \\ \end{array} } \right]$$, $$\Gamma = \left[ {\begin{array}{*{20}l} {\left[ {\nu_{{_{{{\text{ref}}}} }} - 0.4,\nu_{{_{{{\text{ref}}}} }} + 0.4} \right]} \hfill \\ {\left[ { - 0.4, 0.4} \right]} \hfill \\ \end{array} } \right]$$,$${\text{E}} = \left[ {\begin{array}{*{20}c} { - 0.4} \\ { - 0.1} \\ \end{array} } \right]$$,$${\text{H}} = \left[ {\begin{array}{*{20}c} {0.4} \\ {0.1} \\ \end{array} } \right]$$.

The parameters in the aforementioned formulas are described in Table 2.

**Table 2:** Employed model parameters.

Parameters	Description	Parameters	Description
$\varepsilon$	State quantity	$\rho$	The relaxation factor
$u$	Control quantity	$\lambda$	The relaxation coefficient
*e*_*d*_	The distance between P_1_ and M	*R*	The steering radius of vehicle’s rear wheel
*e*_*φ*_	The heading angle deviation	$\kappa$	The steering curvature of vehicle
*M*	The vehicle’s rear axle center	$\Delta {\text{X}}$	The component of the vehicle’s longitudinal path increment in the X-axis of inertial coordinate
*P*_*1*_	The projection of the vehicle’s rear axle center M on the road center line	$\Delta {\text{Y}}$	The component of the vehicle’s longitudinal path increment in the Y-axis of inertial coordinate
$\varphi_{{{\text{ref}}}}$	The angle between the road’s tangent line and the X-axis of the inertial coordinate	*Q, P*	Weight matrices
*φ*	The yaw angle of vehicle	$N$	Predictions horizon
$\kappa_{{{\text{ref}}}}$	The road curvature at point P_1_	$\nu_{\chi }$	The longitudinal velocity of the vehicle in the vehicle coordinate system
*P*_*0*_	A reference point on the center line of the road	$\nu_{\gamma }$	The lateral velocity of the vehicle in the vehicle coordinate system
$\omega$	The yaw angler velocity of vehicle	${\dot{\text{S}}}$	The speed of P_1_ along the road’s center line
${\text{S}}$	The arc length between point P_1_ and P_0_		

### Trajectory tracking scheme design

Several trajectory tracking methods have been proposed^29–31^. However, these methods often struggle to ensure both tracking accuracy and real-time performance at the same time. In this section, we introduce the trajectory Tracking Scheme based on MPC. The new control approach can significantly improve trajectory tracking accuracy, all while ensuring real-time calculations.

According to the analysis in Section "Trajectory tracking method based on MPC", the unmanned vehicle’s control accuracy is restricted by the vehicle’s nonlinear kinematics. When vehicles navigate complex curved roads, significant tracking deviations may occur. To address this issue, a novel control approach for trajectory tracking is proposed in this work. In the new control approach, vehicles choose different model discretization methods based on MPC according to the road curvature. The linear prediction model is used to predict and control the vehicle's trajectory when driving on roads with straight sections or small curvatures. On the other hand, the nonlinear prediction model is used to predict and control the vehicle's trajectory on roads with significant curvatures. This method can combine the advantages of LMPC and NMPC to achieve real-time and accurate trajectory tracking.

**Figure 4:**
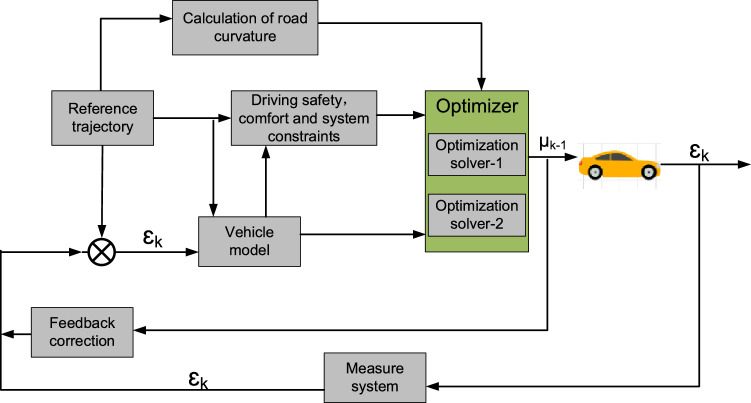
Structure diagram for the MPC scheme.

Figure 4 depicts the suggested MPC scheme's framework. The vehicle model, system restrictions, and the optimizer make up the three blocks of the MPC module. At step k, the measured state $\mathcal{E}$_k_ is denoted as the initial state and the predicted control value u_k-1_ derived from the previous loop is denoted as the initial control value. Accordingly, the vehicle model is developed. Then, driving safety, comfort, and system constraints are designed to balance tracking accuracy, ride comfort, and vehicle maneuverability. Finally, based on the road curvature calculation result, an optimization solver is selected to calculate the control sequence $\upmu$_k_ according to the road curvature. Optimization solver-1, corresponding to LMPC, is selected on highways with straight stretches or mild curves. On the other hand, optimization solver-2, corresponding to NMPC with a higher tracking accuracy, is selected on largely curved roads. The road curvature is calculated from the coordinates of the test road. This predictive control algorithm improves real-time performance and tracking precision at the same time. Further details regarding the optimization solver selection can be found in Section "Simulations and analysis".

## Simulations and analysis

### Simulation description

**Figure 5:**
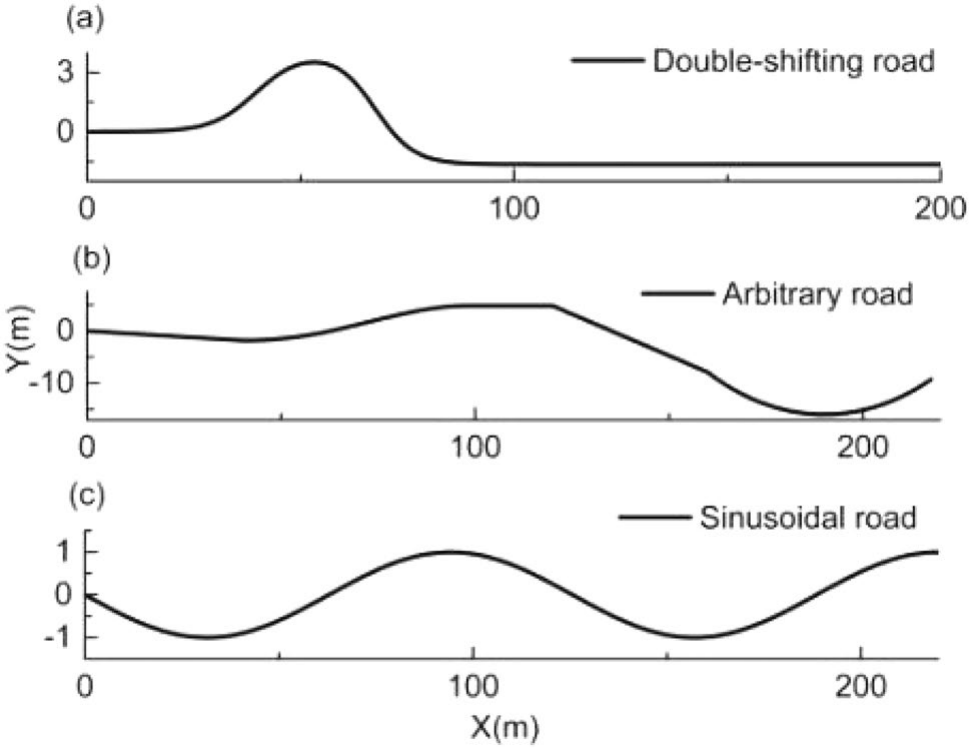
Test road.

MATLAB2020 and CarSim2019 were used as the simulation tools, and a B class Hatchback with rear-wheel drive was used as the controlled unmanned vehicle. The test road coordinates including double-shifting road, arbitrary road, and sinusoidal road, are depicted in Fig. 5. The quadprog solver^32^ is used for linear model predictive control on roads with small curvature. On the other hand, the fmincon solver^33^ is used for nonlinear model predictive control on roads with large curvature. The values of MPC controller parameters are shown in Table 3.

**Table 3:** MPC controller parameters values.

Parameters	Np	Nc	T (s)
Values for LMPC	20	30	0.1
Values for NMPC	10	2	0.03

### Controller selection

To verify the control accuracy and computational efficiency of the proposed predictive control algorithm, we designed three MPC controllers to compare the tracking performance on the test road:

- *Controller I* MPC controller with a linear prediction model.
- *Controller II* MPC controller with nonlinear prediction model.
- *Controller III* MPC controller combining both linear and nonlinear prediction models, as proposed in this work.

The control results from the MPC controllers I and II are analyzed under various speed conditions and road curvatures on the double-shifting road. Based on the analysis, controller III is used to enhance the tracking performance. The simulation results for MPC controller III are presented in Section "Simulations and analysis".

In order to confirm whether the suggested approach can be used to track different trajectories, the tracking results of controllers I, II, and III are compared when tracking arbitrary curves and sinusoidal trajectories. The switching curvature for different solvers of controller III is determined based on the tracking results from the double-shifting road, as detailed in Section "Simulations and analysis".

### Performance evaluation when tracking double-shifting line

#### Performance evaluation using a single algorithm

To examine how road curvature and vehicle speed affect tracking accuracy, we tested the tracking performance of controller I and controller II when the vehicle speed was 1 m/s and 2 m/s. The influence of road curvature on tracking accuracy was also studied.

**Figure 6:**
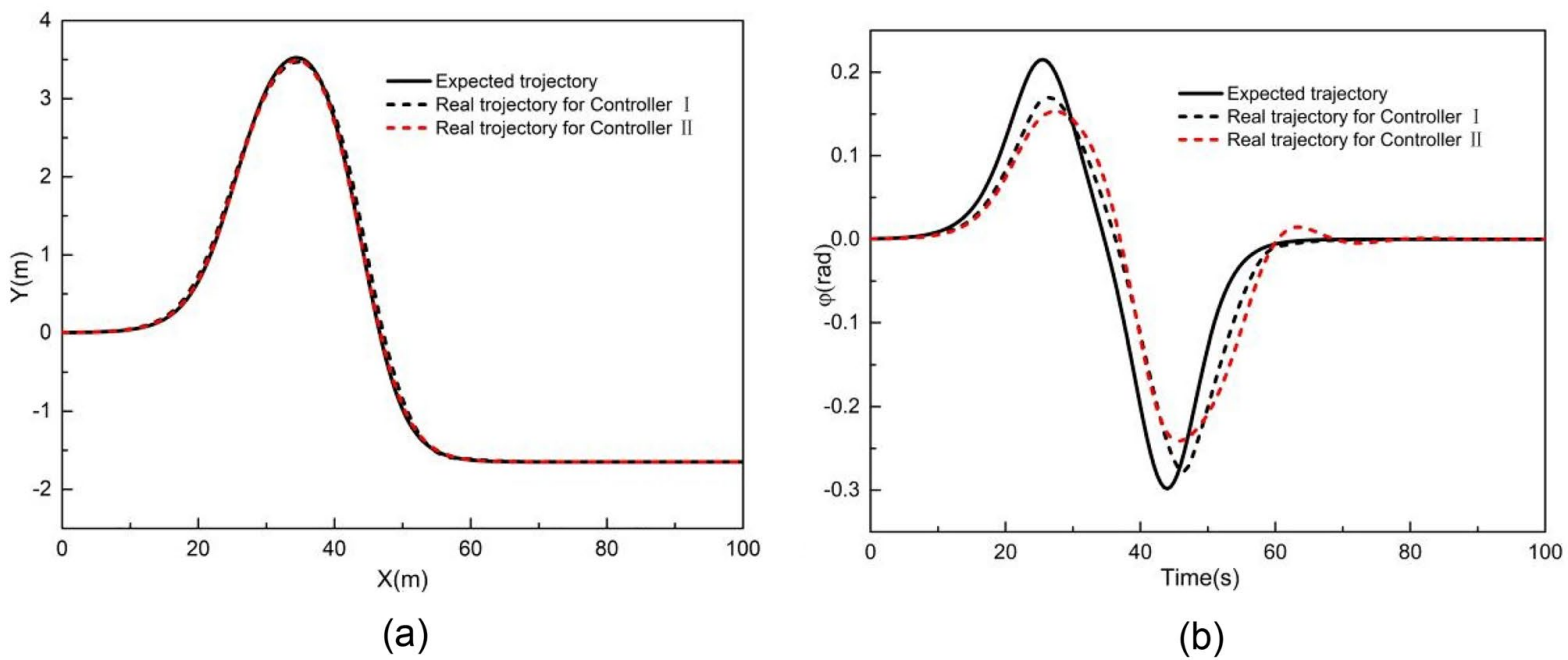
Comparison of (**a**) XY station and (**b**) heading angle at 1 m/s.

**Figure 7:**
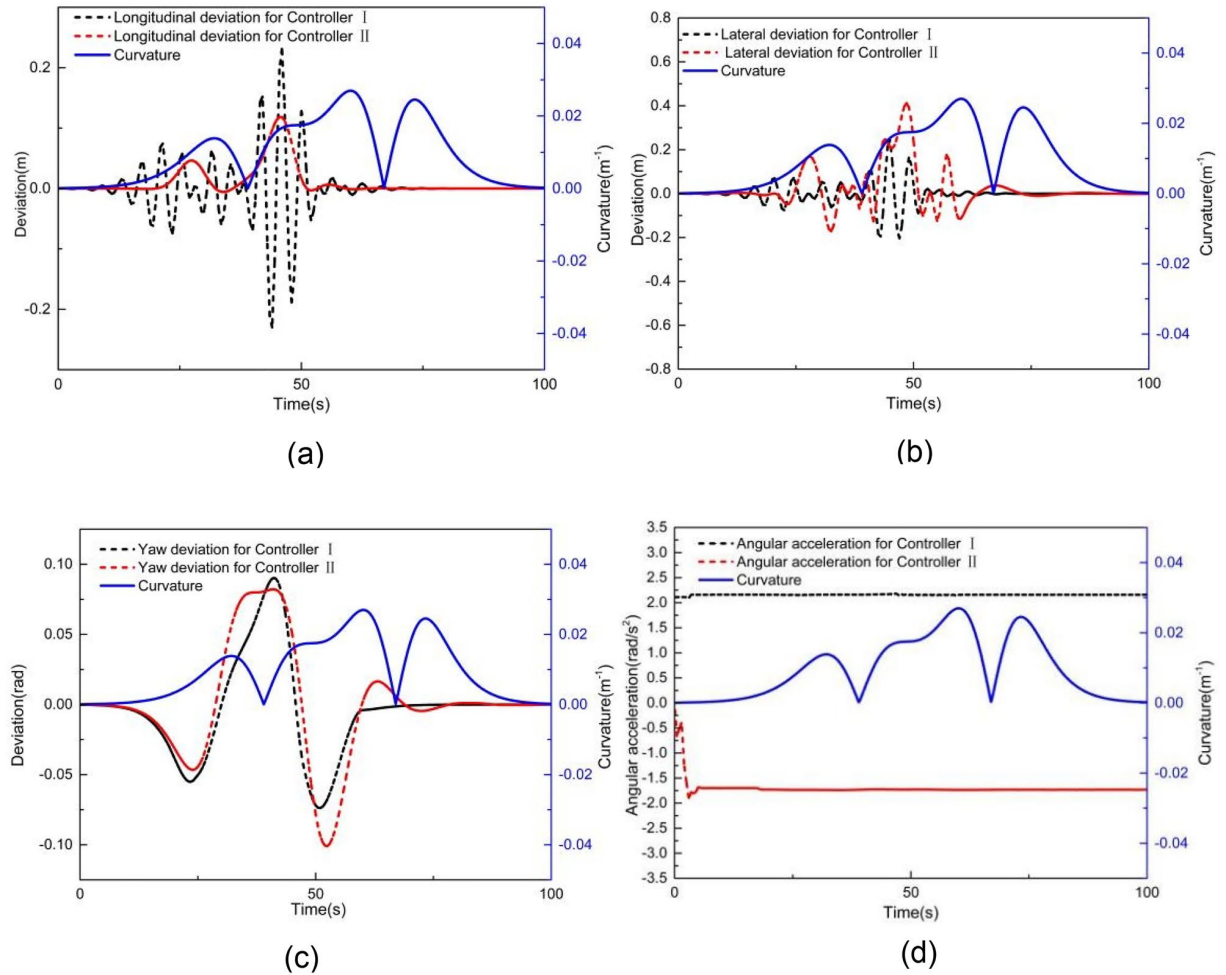
Deviation of (**a**) longitudinal position, (**b**) lateral position, (**c**) heading angle at 1 m/s considering road curvature, and (d) is the angular acceleration at 1 m/s considering road curvature.

When the vehicle speed is 1 m/s, the comparative results with controller I and controller II are shown in Figs. 6 and 7. As shown in Fig. 6, the X station, Y station, and yaw angle are close to the corresponding reference value. Figure 7 presents the tracking deviation of the longitudinal trajectory, lateral trajectory, yaw angle, and angular acceleration, considering the road curvature.

It can be observed that the tracking deviation of controllers I and II is small. When using controller I, the maximum absolute longitudinal deviation is 0.23 m, the maximum absolute lateral deviation is 0.24 m, the yaw angle's maximum absolute deviation is 0.09 rad, and the maximum angular acceleration is 2.16 rad/m^2^. When using controller II, the maximum absolute deviation of longitudinal position, lateral position, and yaw angle are 0.10 m, 0.41 m, and 0.10 rad, respectively. The maximum angular acceleration is 1.90 rad/s^2^. The tracking deviation of controllers I and II is almost the same. In addition, the road curvature has little effect on tracking deviation when the vehicle speed is 1 m/s, to some extent, particularly for the Y station and heading angle.

**Figure 8:**
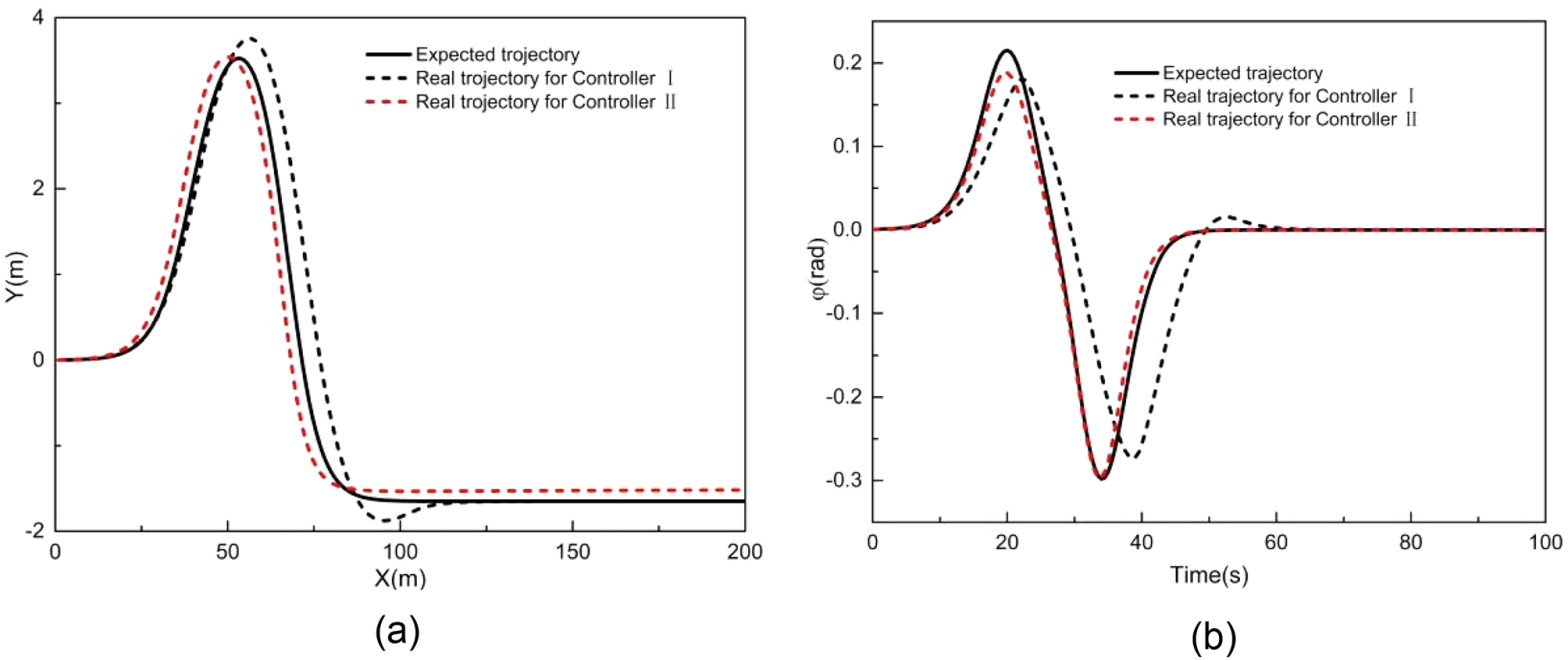
Comparison of (**a**) XY station and (**b**) heading angle at 2 m/s.

**Figure 9:**
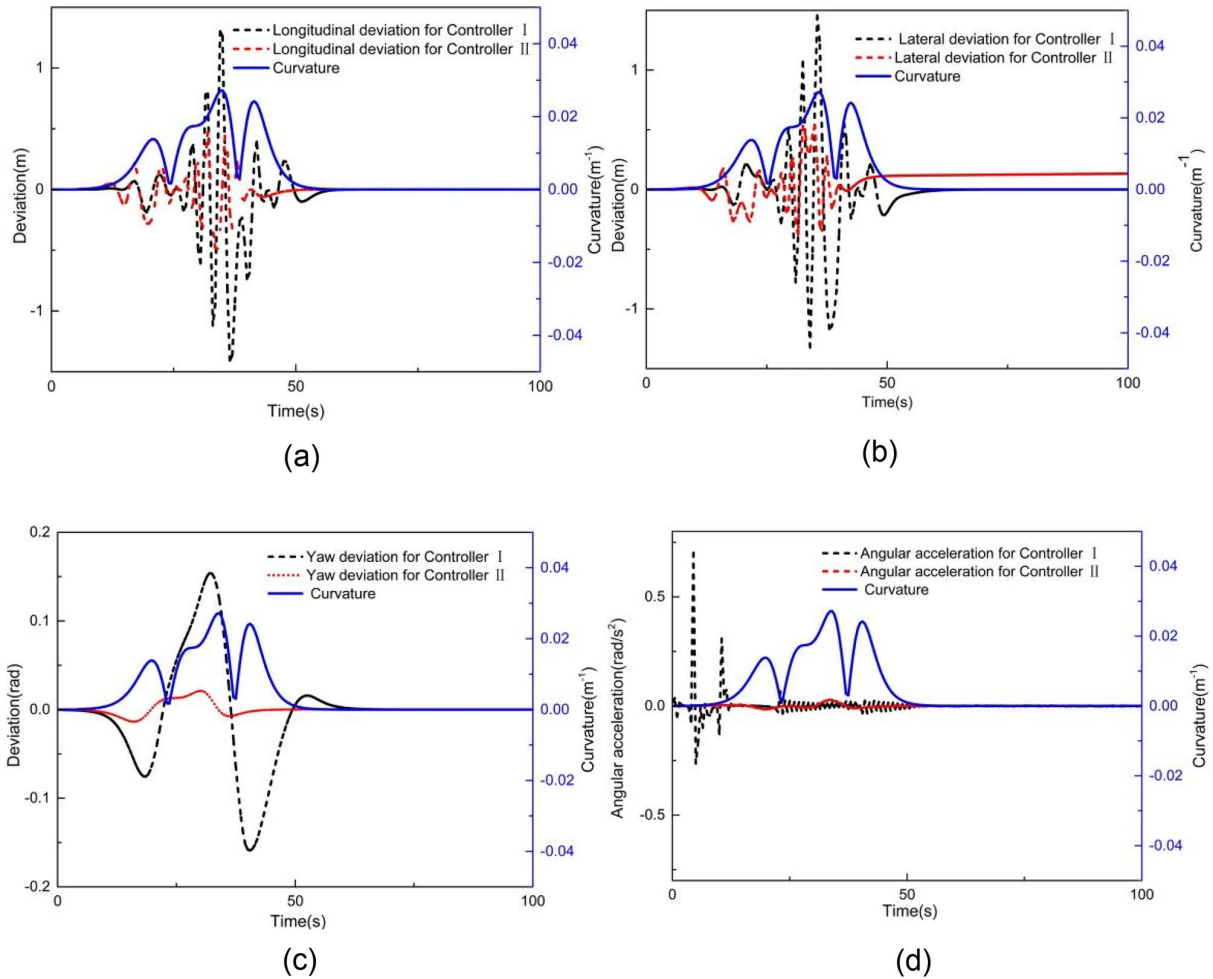
Deviation of (**a**) longitudinal position, (**b**) lateral position, (**c**) heading angle at 2 m/s considering road curvature, and (d) is the angular acceleration at 2 m/s considering road curvature.

Figures 8 and 9 present the simulation comparison results for MPC controllers I and II at 2 m/s. As shown in Fig. 8, both controllers deviate from the expected trajectory. Figure 9 illustrates the tracking deviation of the longitudinal position, lateral position, and heading angle considering the road curvature. The angular acceleration considering the road curvature is also shown in Fig. 9d. It is evident that when using controller I, the maximum absolute deviation of longitudinal position, lateral position and yaw angle are 1.41 m, 1.46 m and 0.16 rad, respectively. The maximum angular acceleration for controller I is 0.70 rad/m^2^. When using controller II, the maximum absolute deviations of longitudinal position, lateral position, and yaw angle are 0.49 m, 0.53 m and 0.29 rad, respectively. The maximum angular acceleration for controller II is 0.028 rad/m^2^.

These results clearly indicate that controller II’s tracking accuracy is significantly greater than that of controller I. Moreover, the yaw acceleration of controller II is notably less than that of controller I, indicating a substantial improvement in the ride comfort for unmanned vehicles when using controller II. Additionally, it can be observed from Fig. 9 that the tracking errors of controllers I and II are small at the beginning of the simulation where the road curvature is small. However, as the simulation progresses, the longitudinal, lateral, and yaw angle deviations for controller I significantly increase with the increase in road curvature. In contrast, although the longitudinal, lateral, and yaw angle deviations for controller II also change with the increase in road curvatures, their increments are comparatively smaller. Notably, the longitudinal and lateral deviations of controller I exceed the maximum absolute error of controller II at 29 s when the road curvature is 0.017 m^−1^. Furthermore, when the simulation time exceeds 60 s and the road curvature returns to 0, the longitudinal, lateral, and yaw angle deviations of controller II are all very close to 0, whereas the lateral deviation of controller I is still larger than that of controller II. Therefore, the tracking accuracy of controller I is better for smaller road curvature, while the tracking accuracy of controller II is better for larger road curvature.

#### Performance evaluation using multiple algorithms

**Figure 10:**
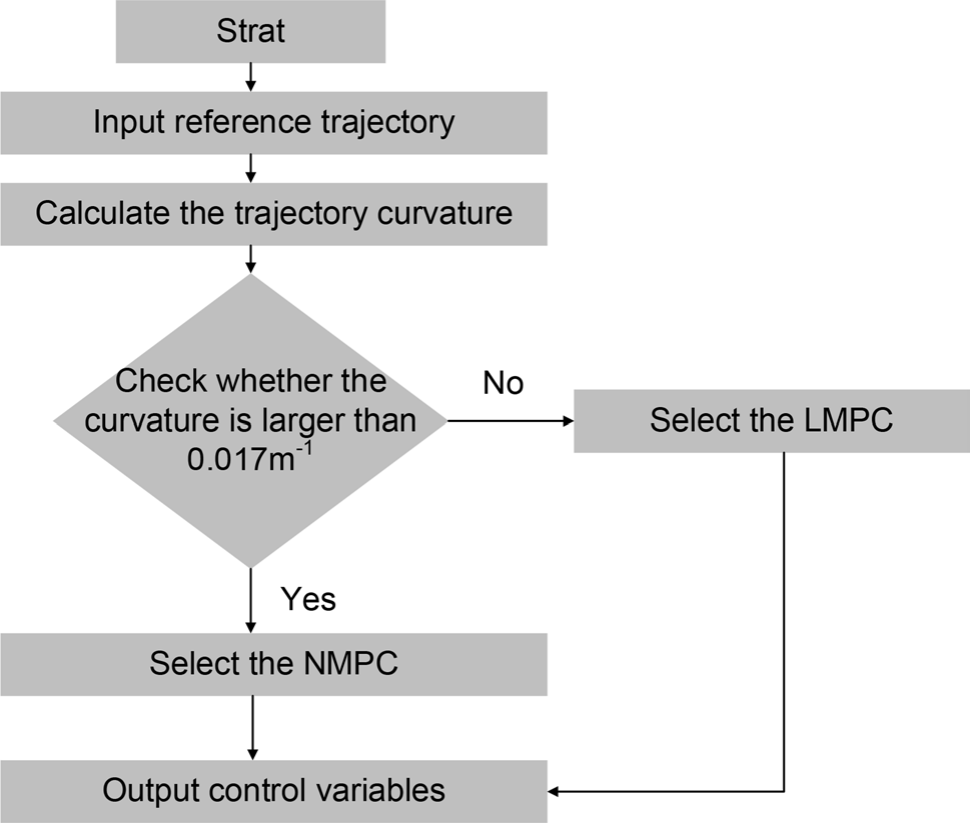
Logic diagram of controller III.

Based on the previous analysis, we have designed controller III, which can switch algorithms according to the road curvature. Figure 10 shows the logic diagram. From Fig. 3, if the road curve is expressed by Eq. (44), the referenced road curve can be calculated through Eq. (45).

44 $$\left\{ {\begin{array}{*{20}c} {{\text{X}} = {\text{f}}_{1} \left( t \right)} \\ {{\text{Y}} = {\text{f}}_{2} \left( t \right)} \\ \end{array} } \right.$$

45 $$\left\{ {\begin{array}{*{20}l} {\varphi_{{{\text{ref}}}} = \arctan \left( {\left. {{\dot{f}}_{2} \left( t \right)/{\dot{f}}_{1} \left( t \right)} \right)} \right.} \hfill \\ {\kappa_{ref} = \dot{\varphi }_{{ref}}/v_{ref} } \hfill \\ \end{array} } \right.$$

Building upon the double-shifting performance in Fig. 9, the longitudinal and lateral deviations of controller I exceed the maximum absolute error of controller II at 29 s when the road curvature is 0.017 m^−1^. Thus, controller III adopts a selection mechanism: if the road curvature is less than 0.017 m^−1^, LMPC is chosen; otherwise, NMPC is employed.

**Figure 11:**
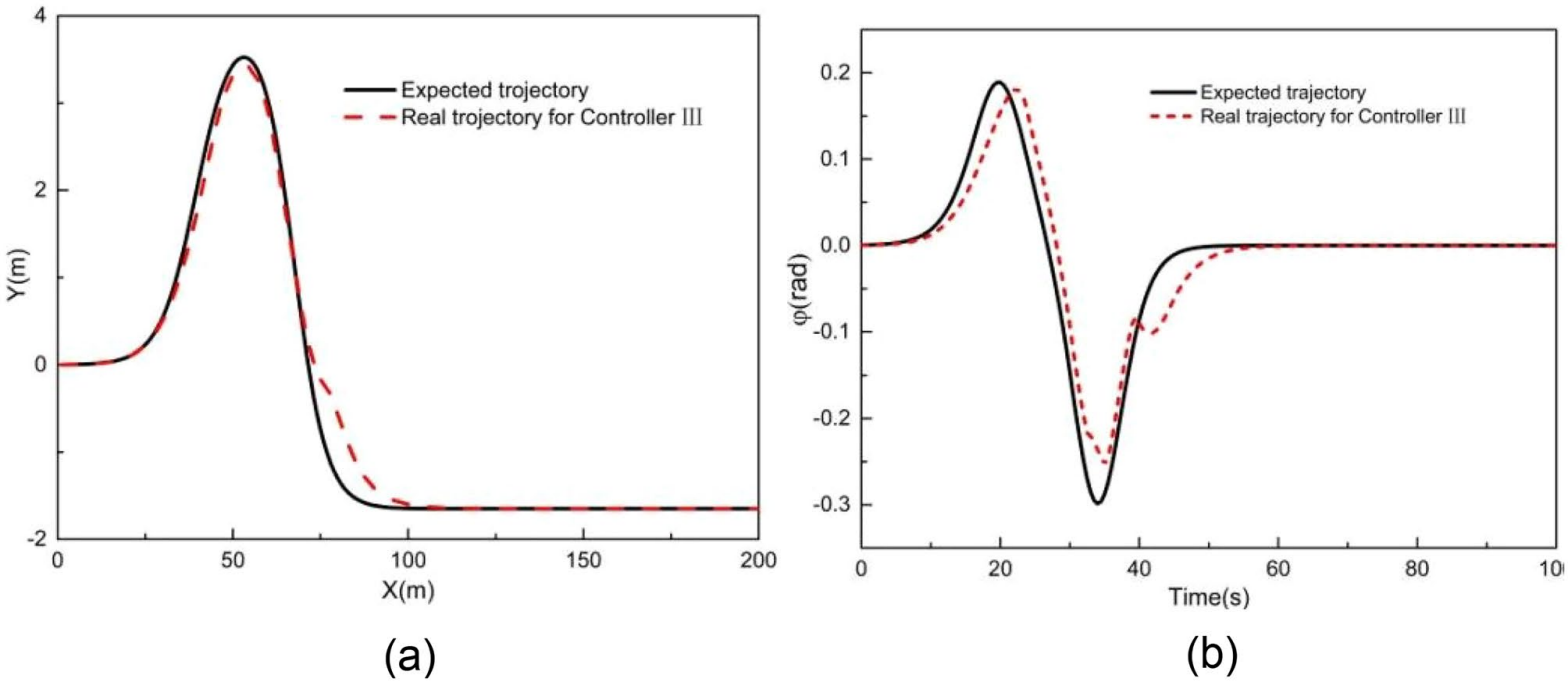
Tracking result of (**a**) XY station and (**b**) heading angle at 2 m/s using controller III.

**Figure 12:**
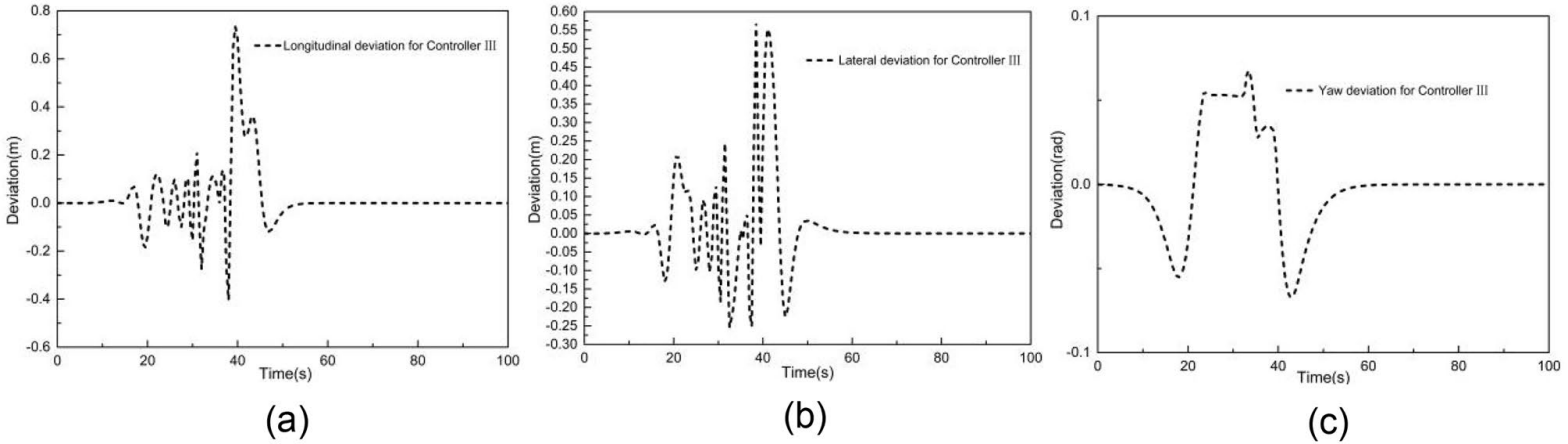
Deviation of (**a**) longitudinal position, (**b**) lateral position, (**c**) heading angle at 2 m/s using controller III.

Figures 11 and 12 illustrate the tracking results with MPC controller III when the vehicle speed is 2 m/s. As shown in Fig. 11, the Y station and the yaw angle are in close alignment with the desired trajectory. Figure 12 displays the tracking deviation of longitudinal trajectory, lateral trajectory, yaw angle, and angular acceleration for controller III.

It can be observed that the maximum absolute deviation of longitudinal position, lateral position, and yaw angle are 0.68 m, 0.57 m, and 0.07 rad, respectively. These maximum absolute deviations are smaller than those of controller I. Besides, throughout the entire simulation time, the tracking deviation remains within a small range. The tracking deviation for controller III combines the advantages of controllers I and II throughout the simulation time, resulting in improved overall tracking performance.

**Figure 13:**
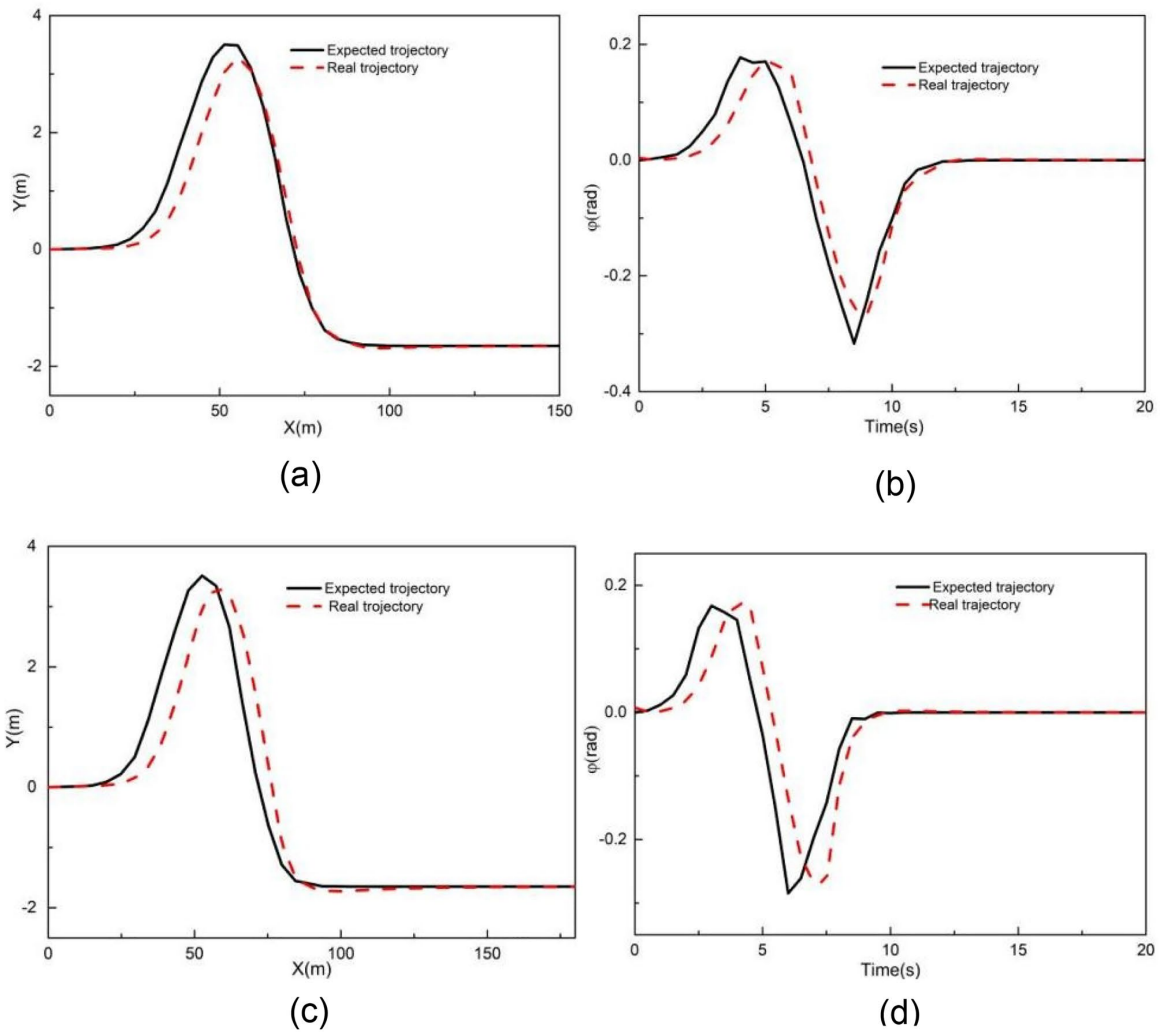
Tracking result of (**a**) XY station at 8 m/s, (**b**) heading angle at 8 m/s, (**c**) XY station at 10 m/s and (**d**) heading angle at 10 m/s using controller III.

**Figure 14:**
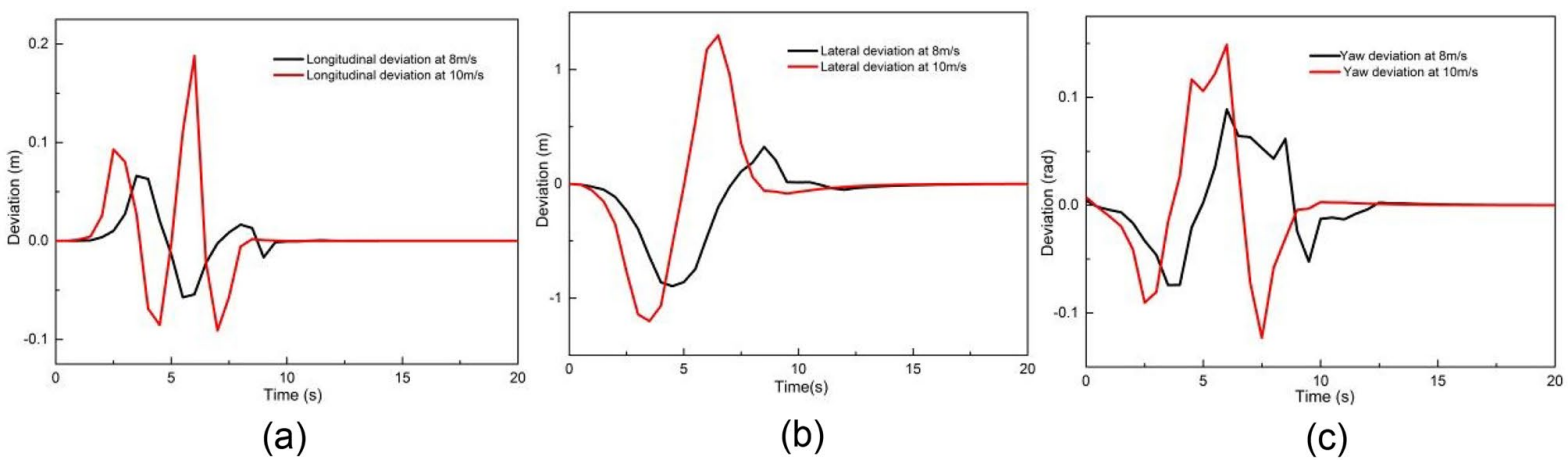
Deviation of (**a**) longitudinal position, (**b**) lateral position, (**c**) heading angle at 8 m/s and 10 m/s using controller III.

Figures 13 and 14 illustrate the tracking results with MPC controller III at longitudinal speeds of 8 m/s and 10 m/s. As shown in Fig. 13, when the longitudinal speed are 8 m/s and 10 m/s, both Y and PHI closely follow the expected values at longitudinal speeds of 8 m/s and 10 m/s. Figure 14 presents the tracking deviations of longitudinal trajectory, lateral trajectory, and yaw angle for controller III at 8 m/s and 10 m/s. It can be seen that as the longitudinal velocity increases, the deviations also increase. The maximum absolute deviations of longitudinal position, lateral position, and yaw angle at 8 m/s are 0.07 m, 0.89 m, and 0.09 rad, respectively. The maximum absolute deviations of longitudinal position, lateral position, and yaw angle at 10 m/s are 0.19 m, 1.29 m, and 0.15 rad, respectively. This may result from unmodeled uncertainties in the kinematic model.

## Real-time performance

**Figure 15:**
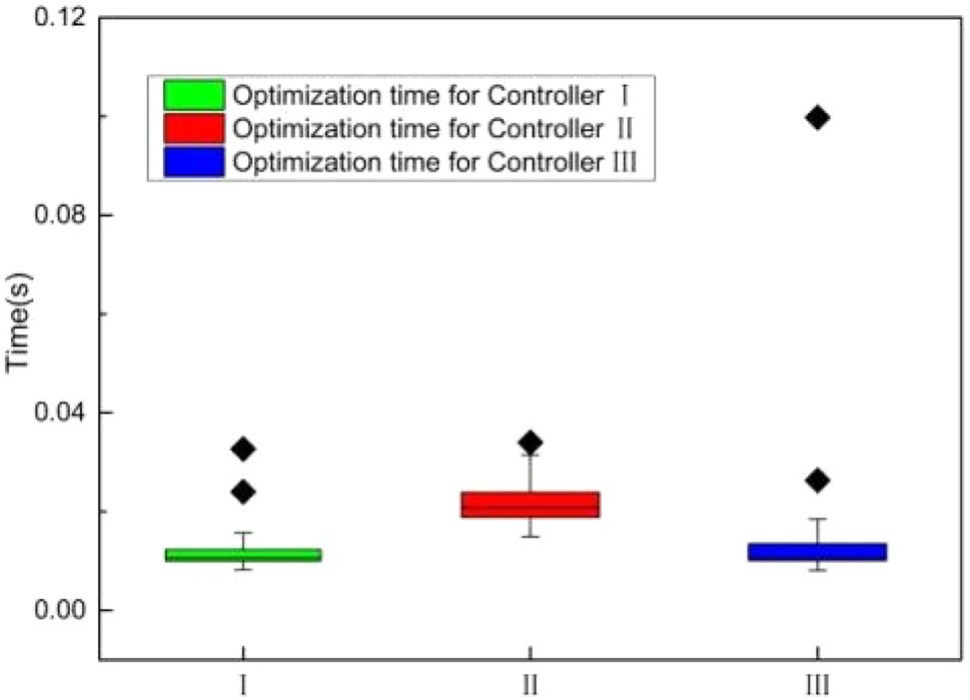
Comparison of the optimization time.

The three designed controllers’ mean optimization time and their standard deviation, at 2 m/s, are depicted in Fig. 15. Controller III's average optimization time is 0.013 s, which is considerably less than controller II's and somewhat greater than controller I's. This suggests that for controller III, the optimization time is comparatively constant. As a result, the proposed MPC scheme is well-suited for real-time applications.

### Performance evaluation using Stanley algorithm

**Figure 16:**
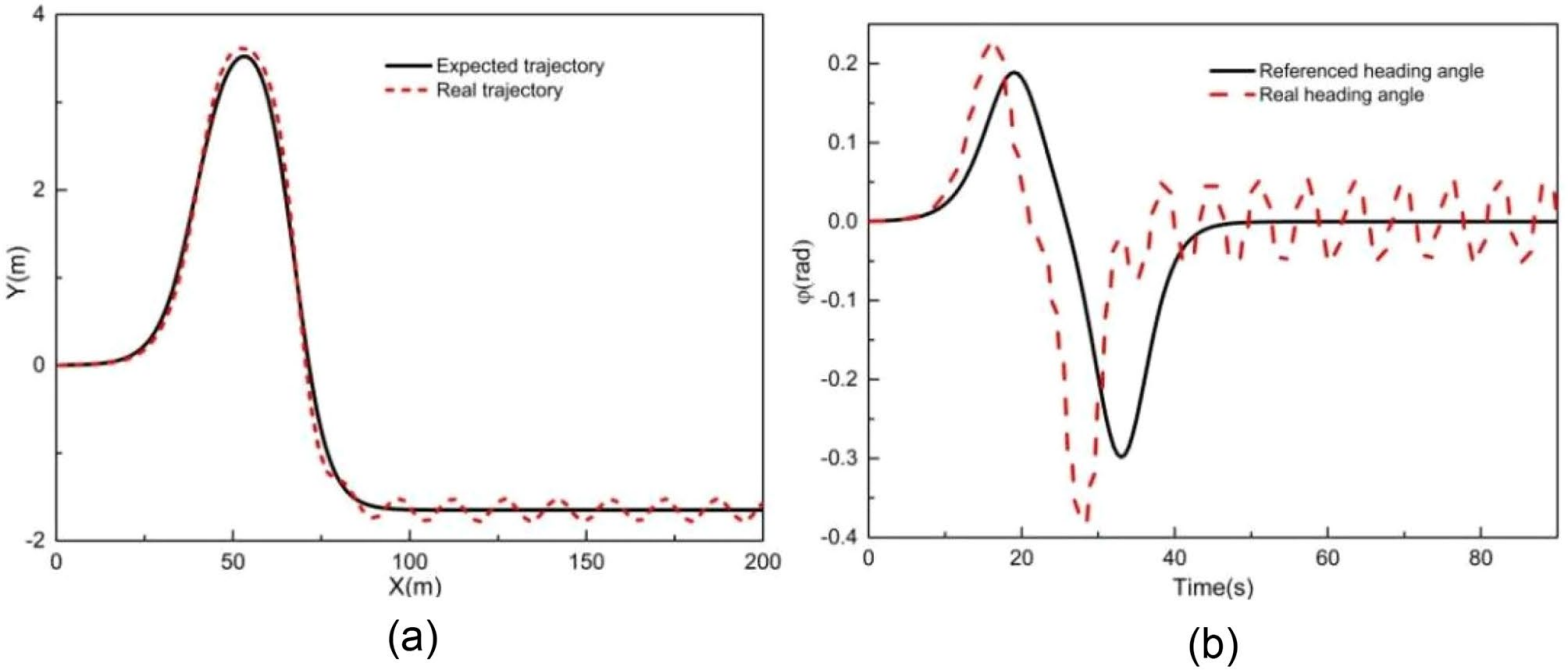
Tracking result of (**a**) XY station and (**b**) heading angle at 2 m/s using Stanley algorithm.

**Figure 17:**
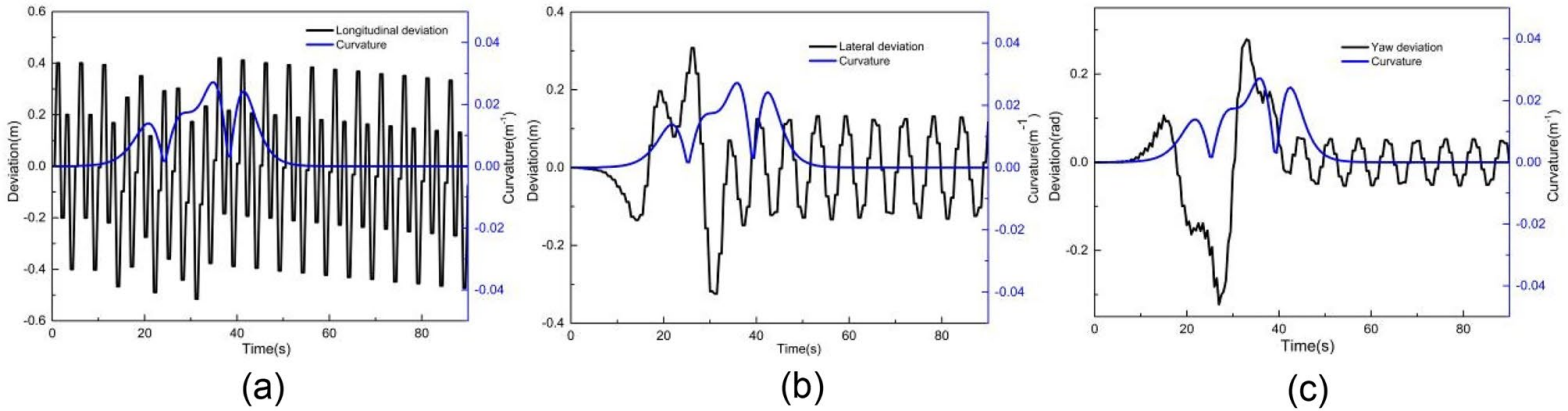
Deviation of (**a**) longitudinal position, (**b**) lateral position, (**c**) heading angle at 2 m/s using Stanley algorithm.

To demonstrate the capability of the proposed scheme to address both tracking accuracy and real-time performance, we use Stanley algorithm to track the double-shifting line at a target longitudinal speed of 2 m/s. Figures 16 and 17 illustrate the tracking results with Stanley algorithm at a vehicle speed of 2 m/s. As illustrated in Fig. 16, there are certain deviations between the Y station and the yaw angle with respect to the desired trajectories. Figure 17 presents the tracking deviations of longitudinal trajectory, lateral trajectory, and yaw angle for Stanley algorithm. It can be observed that the maximum absolute deviations of longitudinal position, lateral position, and yaw angle are 0.51 m, 0.32 m, and 0.32 rad, respectively. These maximum absolute deviations are larger than those of controller III. In addition, the trajectory jitters occur after the tracking time of 30 s. The average computation time for the Stanley algorithm is 6E−4 s, which is slightly shorter than that of Controller III's. These results indicate that the proposed method significantly improves trajectory tracking accuracy, all while ensuring real-time calculations.

### Performance evaluation when tracking an arbitrary curve

**Figure 18:**
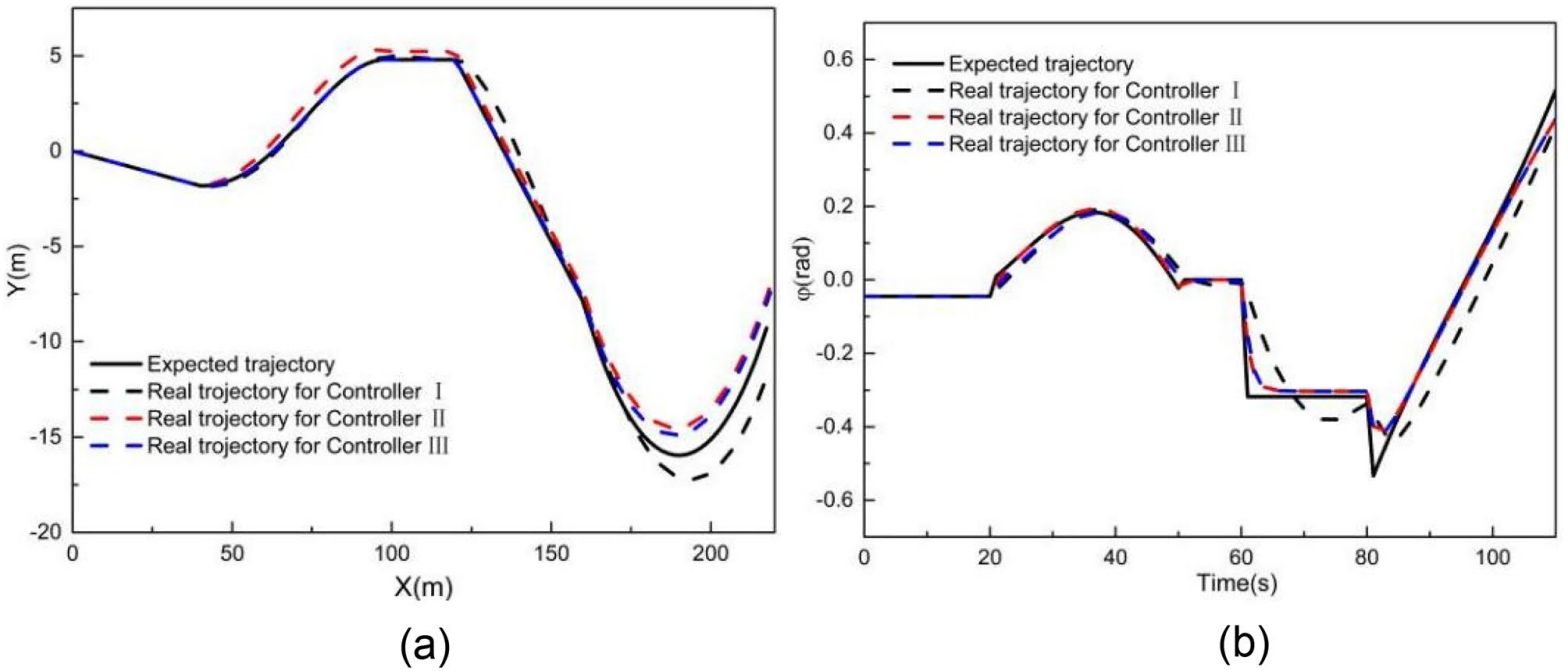
Comparison result when tracking an arbitrary curve at 2 m/s (**a**) XY station and (**b**) heading angle.

**Figure 19:**
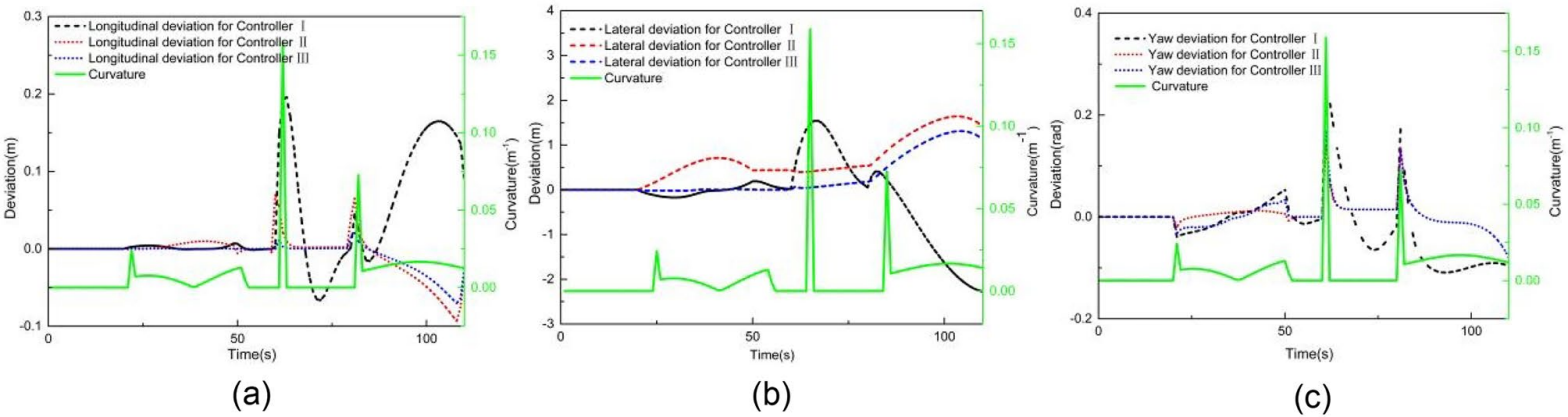
Deviation of (**a**) longitudinal position, (**b**) lateral position, (**c**) heading angle at 2 m/s when tracking an arbitrary curve.

Figures 18 and 19 illustrate the tracking results of different controllers when the vehicle speed is 2 m/s on an arbitrary road. Building upon the double-shifting performance, controller III adopts a selection mechanism: if the road curvature is less than 0.017 m^−1^, LMPC is chosen; otherwise, NMPC is employed.

As shown in Fig. 18, both controllers I and II exhibit some deviation in Y station and heading angle from the expected trajectory, whereas controller III achieves better tracking accuracy, with the Y station and yaw angle closely aligned with the desired trajectory.

Figure 19 provides a comparison of tracking deviations in longitudinal trajectory, lateral trajectory, and yaw angle. When using controller I, the maximum absolute deviation of longitudinal position, lateral position, and yaw angle are 0.19 m, 2.27 m, and 0.28 rad, respectively. When using controller II, these values improve to 0.09 m, 1.64 m, and 0.14 rad, respectively. However, when using controller III, the maximum absolute deviations of longitudinal position, lateral position, and yaw angle further reduced to 0.07 m, 1.30 m, and 0.17 rad, respectively. These results demonstrate that controller III outperforms both controllers I and II in terms of tracking accuracy on arbitrary-curve roads.

### Performance evaluation when tracking a sinusoidal curve

**Figure 20:**
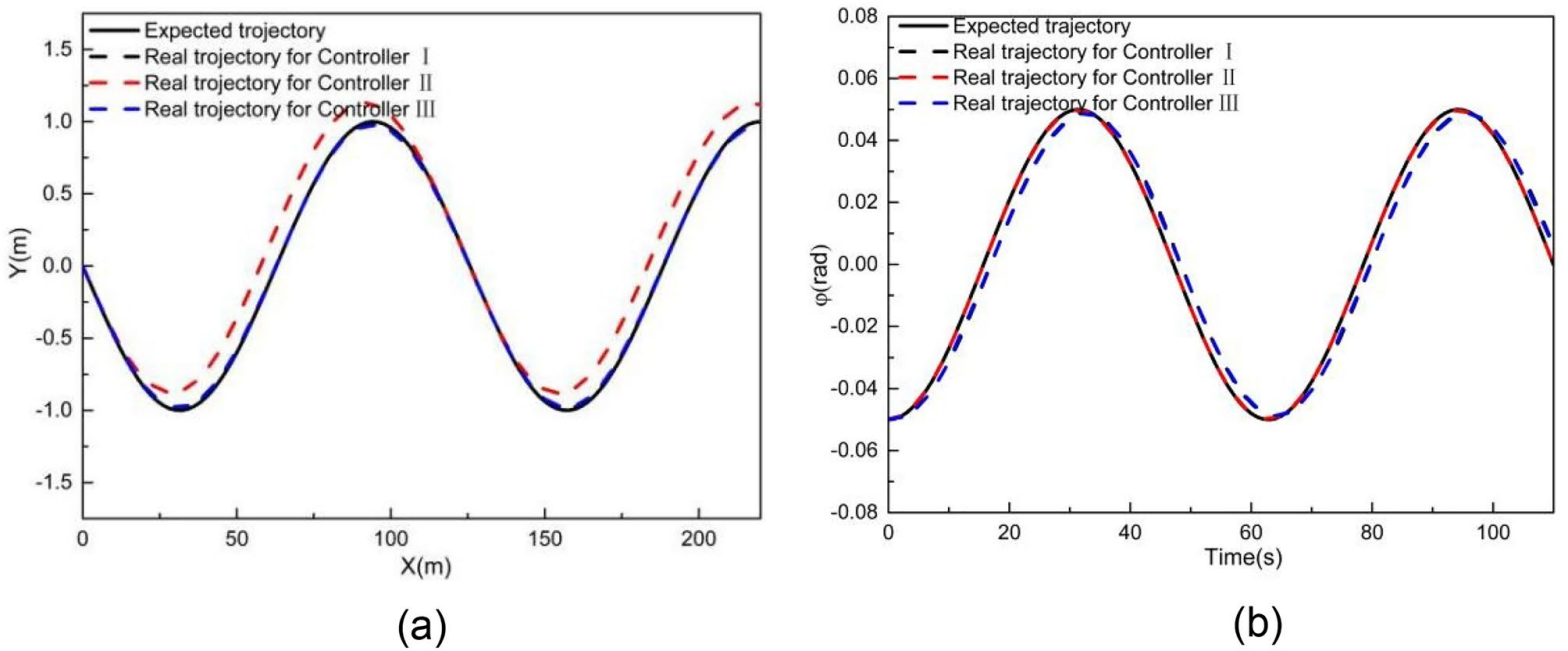
Comparison result when tracking a sinusoidal curve at 2 m/s (**a**) XY station and (**b**) heading angle.

**Figure 21:**
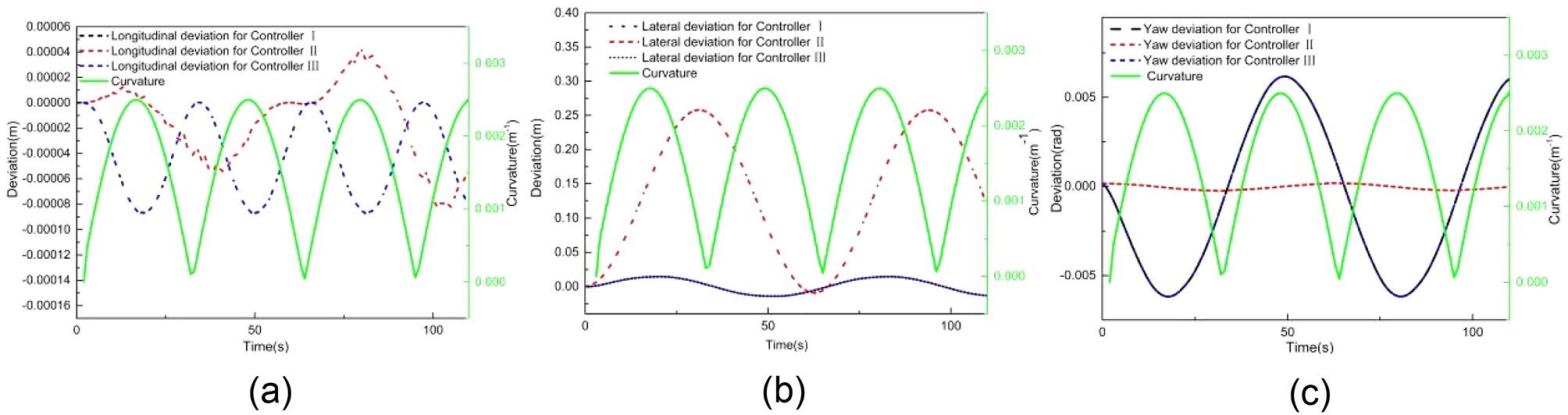
Deviation of (**a**) longitudinal position, (**b**) lateral position, (**c**) heading angle at 2 m/s when tracking a sinusoidal curve.

Figures 20 and 21 show the tracking results, at 2 m/s, on a sinusoidal road. As shown in Fig. 20, controller II exhibits some deviation in Y station and heading angle from the expected trajectory. However, controllers I and III achieve better tracking accuracy, with the Y station and yaw angle closely following the desired trajectory.

Figure 21 provides a comparison of tracking deviations in longitudinal trajectory, lateral trajectory, and yaw angle. As the curvature of the entire sinusoidal road is smaller than 0.017 m^−1^, controllers I and III exhibit the same tracking deviations. When using these controllers, the maximum absolute deviations of longitudinal position, lateral position, and yaw angle are 8.68E − 5 m, 0.014 m, and 0.006 rad, respectively. When using controller II, the maximum absolute deviation of longitudinal position, lateral position, and yaw angle are 2.6E − 4 m, 0.26 m, and 0.00026 rad, respectively. The result of Fig. 21 shows that controller III can still guarantee the tracking accuracy when the road curvature is small. Therefore, in this case, the tracking accuracy can also be guaranteed through intelligent selection of MPC algorithm.

## Conclusion

In this paper, we analyzed the effects of road curvature and vehicle speed on tracking accuracy using both LMPC and NMPC algorithms under the double line shifting condition. Based on the analysis results, we introduced a new trajectory tracking method to improve tracking accuracy. The feasibility of the new method was verified on different testing roads.

The key conclusions drawn from this study are as follows:

1. At a vehicle speed of 1 m/s, both the LMPC and NMPC demonstrate good tracking performance with very small tracking deviation. Additionally, the road curvature does not significantly affect the tracking accuracy at this speed.
2. At a vehicle speed of 2 m/s, LMPC shows better tracking accuracy when the road curvature is small, while NMPC performs better when the road curvature is large.
3. The proposed trajectory tracking method significantly improves tracking accuracy at a vehicle speed of 2 m/s, while maintaining a comparable calculation time to LMPC. This finding suggests that the proposed method achieves a desirable balance between tracking accuracy and computational efficiency.
4. The NMPC algorithm based on tracking errors in this work considers the effect of road curvature and vehicle curvature on tracking accuracy, making it suitable for tracking roads with large curvature. This algorithm greatly simplifies the calculation formula for tracking curved roads compared to the vehicle dynamic model.
5. The proposed method intelligently switches between LMPC and NMPC based on the road curvature, making it applicable to a wide range of complex trajectories.

It should be noted that this work is based on the 2-DOF vehicle model. In the future, we will consider the applicability of this model to the 3-DOF vehicle model and conduct real-vehicle experiments to further verify the effectiveness of the proposed control scheme.

## Data Availability

The data supporting the results reported in the article are available from the corresponding author on reasonable request.
